# GhMYB52 Like: A Key Factor That Enhances Lint Yield by Negatively Regulating the Lignin Biosynthesis Pathway in Fibers of Upland Cotton (*Gossypium hirsutum* L.)

**DOI:** 10.3390/ijms25094921

**Published:** 2024-04-30

**Authors:** Yang Yang, Xue Zhou, Xi Zhu, Bo Ding, Linzhu Jiang, Huiming Zhang, Silu Li, Shuyan Cao, Mi Zhang, Yan Pei, Lei Hou

**Affiliations:** 1 College of Agronomy and Biotechnology, Southwest University, Chongqing 400715, China; neo5334@163.com (Y.Y.); zhuxi0603@email.swu.edu.cn (X.Z.); 15086655435@163.com (B.D.); linzhuhh@163.com (L.J.); zhang15828612304@163.com (H.Z.); lisilu20010817@outlook.com (S.L.); shuyan961029@163.com (S.C.); selenazm@swu.edu.cn (M.Z.); peiyan3@swu.edu.cn (Y.P.); 2 Chongqing Key Laboratory of Application and Safety Control of Genetically Modified Crops, Southwest University, Chongqing 400715, China; 3 Laboratory Animal Center, Southwest University, Chongqing 400715, China; yokie01@swu.edu.cn

**Keywords:** *Gossypium hirsutum* L., R2R3 MYB transcription factor, GhMYB52 Like, secondary cell wall, lignin biosynthesis, phenylpropanoid pathway, cellulose synthesis, CRISPR-Cas9

## Abstract

In the context of sustainable agriculture and biomaterial development, understanding and enhancing plant secondary cell wall formation are crucial for improving crop fiber quality and biomass conversion efficiency. This is especially critical for economically important crops like upland cotton (*Gossypium hirsutum* L.), for which fiber quality and its processing properties are essential. Through comprehensive genome-wide screening and analysis of expression patterns, we identified a particularly high expression of an R2R3 MYB transcription factor, GhMYB52 Like, in the development of the secondary cell wall in cotton fiber cells. Utilizing gene-editing technology to generate a loss-of-function mutant to clarify the role of GhMYB52 Like, we revealed that GhMYB52 Like does not directly contribute to cellulose synthesis in cotton fibers but instead represses a subset of lignin biosynthesis genes, establishing it as a lignin biosynthesis inhibitor. Concurrently, a substantial decrease in the lint index, a critical measure of cotton yield, was noted in parallel with an elevation in lignin levels. This study not only deepens our understanding of the molecular mechanisms underlying cotton fiber development but also offers new perspectives for the molecular improvement of other economically important crops and the enhancement of biomass energy utilization.

## 1. Introduction

The secondary cell wall (SCW) constitutes a major biomass component in terrestrial plants, primarily comprising cellulose, hemicellulose, and lignin [1,2,3]. Elucidating the mechanisms behind SCW formation is essential for comprehending plant cell development, biodiversity, and environmental adaptability [3]. Furthermore, unraveling the regulatory pathways of SCW biosynthesis is crucial for genetically engineering plants to produce novel materials and bioenergy solutions [4,5]. The R2R3 MYB transcription factor subfamily, distinguished by two adjacent MYB binding domains—R2 and R3 repeats [6,7]—plays a central role in plant biology, regulating a broad spectrum of processes ranging from secondary metabolism and cell cycle regulation to biotic and abiotic stress responses. This influence is due to their remarkable diversity and wide functional range [7,8]. They also engage in the complex regulation of SCW biosynthesis, with MYB46 and MYB83 acting as master regulators under the control of the Secondary Wall NAC Master Switch (SWN), as well as MYB20, MYB52, MYB58, and MYB63, directly mediating SCW biosynthesis [9]. The intricate regulatory networks involved in SCW synthesis pose challenges in regard to fully understanding the roles of R2R3 MYBs in this process [9]. The functionality of R2R3 MYBs has been extensively studied in *Arabidopsis thaliana*, yet research on their roles in relation to economically important crops, such as upland cotton (*Gossypium hirsutum*), is still in a nascent phase.

Cotton, one of the most important natural fiber crops in the world, generates fibers from the differentiation of epidermal cells on the seed coat. The development of these fibers unfolds through several distinct yet interconnected stages: initiation, rapid elongation (primary wall formation), secondary wall synthesis, and dehydration maturation. The pronounced phases of primary and secondary cell wall growth unique to cotton fibers allow them to serve as an ideal model for investigating the regulation of SCW synthesis [10,11]. The deposition of the SCW is crucial in determining the mature fibers’ key quality attributes, including strength, fineness, uniformity, and lint weight [12,13]. While MYB52 has been shown to respond to regulatory factors involved in the formation of the SCW [14,15,16], conclusive evidence regarding its influence on cellulose synthesis is lacking. Furthermore, while MYB52 has been related to the lignin biosynthesis pathway, the nature of its regulatory role—whether it acts as an activator or inhibitor—remains a subject of ongoing debate [15,17,18,19]. Notably, mature fibers consist of almost 94% cellulose, making them highly suitable as raw materials for the textile industry, while their lignin content is only about 2.23–2.63% [20], significantly lower than the 25–30% found in the bark or wood of gymnosperms or angiosperms. Increased cellulose content enhances textile dyeing and finishing performance, whereas higher lignin content typically yields less favorable results. However, the mechanisms behind the high cellulose and low lignin content in cotton fibers remain unclear.

In this study, we explored *GhMYB52 Like* in upland cotton, noting its pronounced expression during the key period of SCW development in cotton fibers, indicating its significant role in regulating lignin synthesis. Through detailed analyses of expression patterns, functional validation experiments, and regulation studies of genes associated with cellulose and lignin synthesis, we comprehensively elucidated the role of *GhMYB52 Like* in cotton fiber development. Our investigation not only contributes to the theoretical understanding of the molecular mechanisms underlying cotton fiber quality traits but also provides fresh perspectives on the enhancement of plant biomass energy and the improvement of other economic crops.

## 2. Results

### 2.1. Profiling the Expression Patterns of the R2R3 MYB Gene Family in Upland Cotton

**Figure 1:**
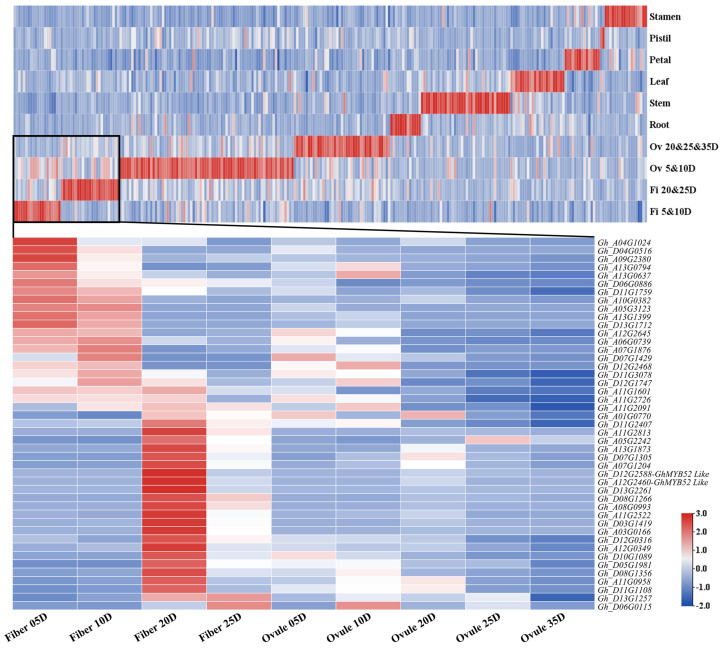
Heatmap of expression patterns of the presumptive R2R3 MYB gene family in *Gossypium hirsutum.* This visualization displays the expression levels of R2R3 MYB genes across different tissues and key developmental stages, with a focus on fiber-specific expression. Expression levels are depicted on a color scale, with the deepest red denoting the highest expression level for each gene in its specific tissue and not the highest absolute expression values across genes. This approach facilitates comparisons of gene expression within tissues and developmental stages rather than between genes. Genes are displayed in the figure only if they achieve an FPKM threshold of 5 or higher in any tissue. The upper portion of the heatmap showcases 269 R2R3 MYB transcription factors that satisfy this threshold. Each row denotes a distinct tissue type, where “Fi” represents fibers, “Ov” signifies ovules, and “05D” refers to 5 days post anthesis. The “&” symbol is used to indicate that the data in a given row were compiled from multiple samples to provide a comprehensive overview. Due to limitations in display resolution, gene IDs are omitted, and each column is dedicated to a different gene. In the lower section, the expression profiles of 45 genes are presented, offering a detailed view of the fiber-dominant-expression R2R3 MYB transcription factors identified from the analysis above. In this section, each row corresponds to an individual gene, while columns map to various tissues.

To gain a comprehensive understanding of the chromosomal distribution and expression patterns of R2R3 MYB gene family members in upland cotton, we performed a genome-wide analysis, identifying 733 genes containing one or more MYB binding domains. Of them, 414 were delineated as R2R3 MYB gene family members (see Table S1). A circle plot revealed that the R2R3 MYBs are most densely distributed on chromosomes A11 and D11, with gene counts of 27 and 25, respectively (as denoted by red vertical lines in Figure S1). The average length of amino acids across these gene family members was calculated to be 311.8. An expression analysis conducted during the secondary cell wall (SCW) formation phase, exemplified by fibers at 20 days post anthesis (DPA), revealed that R2R3 MYBs on chromosomes A05 and A11 exhibited elevated transcriptional activity in this period, as illustrated by green bars in Figure S1. Collinearity analysis, linking genes with high sequence homology (shown by inner circle lines in Figure S1), identified 116 gene pairs connected by a single line, 134 genes connected by multiple lines, and 48 genes unconnected by any other numbers of lines. A multi-tissue digital expression analysis (depicted in the upper half of Figure 1) suggested that a majority (115 members) of the genes in this family exhibit significant expression at various stages of ovule cell development, indicating the necessity of intricate regulatory networks for seed maturation. Furthermore, 26 genes were predominantly expressed in 20 DPA or 25 DPA fiber cells (the lower half of Figure 1, Table S2), suggesting their critical role in the developmental stage of the SCW of fibers.

### 2.2. GhMYB52 Like Preferentialy Expresses during Secondary Cell Wall Deposition of Fiber Cells

**Figure 2:**
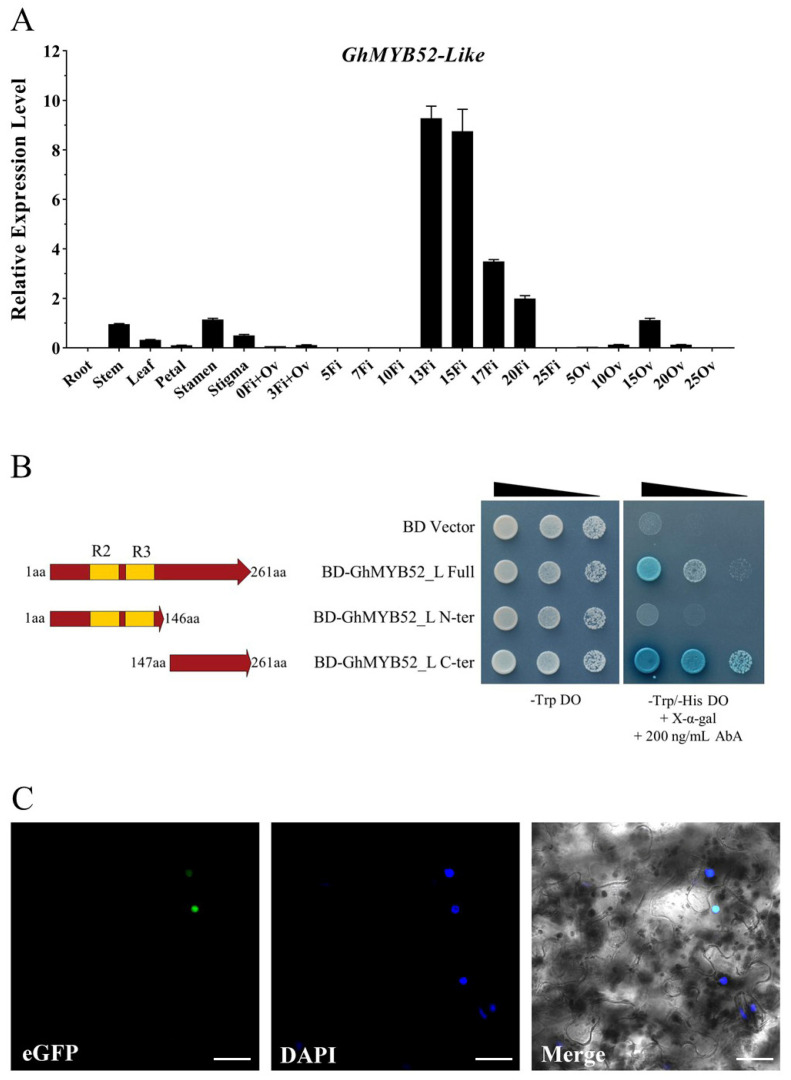
GhMYB52 Like as a transcription factor associated with secondary cell wall development in cotton fiber cells. (**A**) Identification of the relative expression levels of *GhMYB52 Like* in multiple tissues of the wild-type upland cotton HM-1. Numbers denote days post anthesis. “Fi” stands for fibers, “Ov” indicates ovules stripped off fibers, and “Fi+Ov” represents ovules with fibers. Specific primers are capable of amplifying transcripts of the gene pair *Gh_A12G2460* and *Gh_D12G2588*, both of which represent *GhMYB52 Like*. *GhHistone3* was used as the internal reference gene, and each experiment was repeated at least three times. The data highlight the fiber-specific expression of *GhMYB52 Like*. (**B**) Yeast activation verification assays of GhMYB52 Like. Utilizing the yeast two-hybrid system, constructs encoding the full-length and truncated versions of *GhMYB52 Like* were inserted downstream of the BD domain and transformed into Y2H strains to conduct transcriptional activation assays. Strains harboring the BD vector were able to proliferate on media deficient in tryptophan, which served as the fundamental growth control. To assess transcriptional activation potential, media lacking both histidine and tryptophan were utilized, further supplemented with Aureobasidin A (AbA) and X-α-gal. The presence of wedge-shaped markings above the yeast colonies indicates the application of yeast suspensions at varying dilution levels onto the respective agar media. The results indicate that GhMYB52 Like possesses transcriptional activation capability, with the corresponding activation domain located in the C-terminal region. (**C**) Microscopic observation of the subcellular localization of GhMYB52 Like. A fusion protein construct, linking eGFP to the C-terminus of GhMYB52 Like and controlled by the CaMV35S promoter, enabled transient expression in tobacco leaf mesophyll cells. DAPI (4′,6-diamidino-2-phenylindole) was utilized to stain the nuclei of tobacco. Optical signals from both DAPI and eGFP were captured using a laser-scanning confocal microscope. Scale bar: 30 μm.

Using amino acid sequences from the *Arabidopsis* R2R3 MYB gene family, we constructed the phylogenetic tree (Figures S2 and S3). This analysis indicated that AtMYB52, Gh_A11G0016, Gh_D11G0013, Gh_A12G2460, and Gh_D12G2588 form a monophyletic group. Accordingly, *Gh_A11G0016*, and *Gh_D11G0013* were designated as belonging to *GhMYB52*, and *Gh_A12G2460* and *Gh_D12G2588* were designated as belonging to *GhMYB52 Like*. Quantitative real-time PCR analysis conducted across 21 diverse tissues from the wild-type upland cotton cultivar HM-1 revealed that *GhMYB52* exhibited its highest expression in the stem, with notable levels also present during the SCW development stages in fibers. Additionally, this gene showed some expression in ovules between 10 and 35 DPA, while its expression was undetectable in leaves, petals, stamens, and stigmas (Figure S5). Simultaneously, *GhMYB52 Like* exhibited higher transcription levels in fibers during the secondary growth phase, which spans 13–20 DPA (Figure 2A). Although *GhMYB52 Like* is also expressed in 15 DPA ovule cells, stems, leaves, stamens, and stigma tissues, its expression levels are significantly lower than they are in the fiber cells.

To further elucidate the function of MYB52 in cotton fiber cells, GhMYB52 Like, which preferentially expressed during the fiber SCW development phase, was selected as the primary candidate for this study. We successfully cloned the coding sequence of *Gh_D12G2588* from cotton fiber cDNA, which has a CDS length of 786 bp and encodes for 261 amino acids (Figure S4C). Genomic structure analysis revealed that its coding region comprises three exons (Figure S4A). The three-dimensional structure of GhMYB52 Like, predicted using the deep convolutional neural network model AlphaFold2, features the characteristic helix–turn–helix (HTH) structural motif (Figure S4B). Transcriptional activation assays based on the yeast two-hybrid system demonstrated that full-length GhMYB52 Like and GhMYB52 Like C-terminal transformants constructed in the BD vector could grow on a histidine/tryptophan double-deficient solid medium supplemented with Aureobasidin A and turn X-alpha-Gal blue (Figure 2B). These findings indicate that both the full-length GhMYB52 Like and its C-terminal possess transcriptional activation capacity, with the core activation domain located within the last 115 amino acids of the C-terminus. Subcellular localization experiments in which the GhMYB52 Like C-terminal was fused to eGFP showed colocalization with the nuclear stain DAPI (Figure 2C), proving that GhMYB52 Like is a nuclear protein.

### 2.3. Functional Knockout of GhMYB52 Like in Cotton Results in A Decrease in Fiber Yield

**Figure 3:**
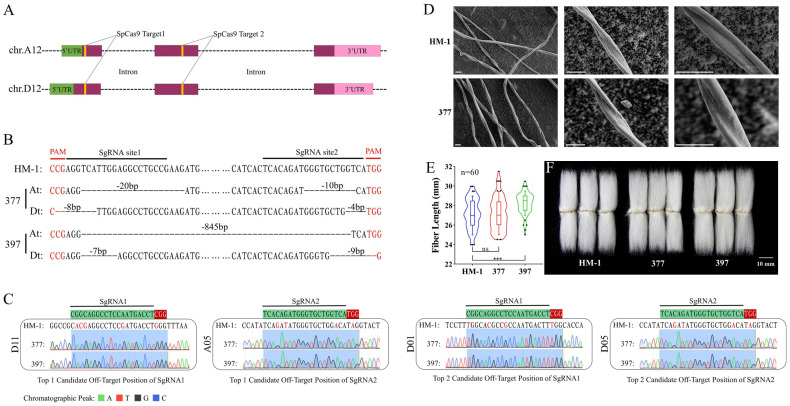
Assessment of CRISPR-Cas9-mediated knockout of *GhMYB52 Like* in cotton and its impact on fiber development. (**A**) Schematic overview of CRISPR-Cas9-targeted sites on chromosomes A12 and D12. The genes *Gh_A12G2460* and *Gh_D12G2588*, encoding GhMYB52 Like, are illustrated with untranslated regions (UTRs) in green and pink, coding exonic regions in burgundy, and with non-coding sequences presented as dashed lines. For genomic editing, two SpCas9 guide RNA sequences were designed, with each targeting specific sites on the two chromosomes (denoted by yellow blocks in the diagram), to achieve targeted modifications within the exonic regions. (**B**) Sanger sequencing identification of the genome-edited sequences in the T_2_ generation transgenic lines 377 and 397. The HM-1 base sequence presented between the yellow blocks in Subfigure A highlights the native, unedited sequences flanking the 20 bp guide sequences. Ellipses are used to denote sections of the sequence that are not fully displayed. Horizontal solid lines indicate the specific guide sequence regions directed by Cas9 towards the *GhMYB52 Like* gene (black line) and the requisite PAM recognition sites (red line). At and Dt denote the A and D sub-genomes, respectively. Black dashed lines depict the regions excised from the A/D subgenomes in lines 377 and 397 as a result of the editing process, with the numerals atop these lines specifying the exact number of bases eliminated. In the T_2_ generation, both sub-genomes of lines 377 and 397 exhibited homozygous mutations. (**C**) Sequencing analysis of potential off-target sites around the genome. For each guide RNA sequence, two top high-probability off-target sites were chosen for Sanger sequencing to assess whether the sequences at these loci in lines 377 and 397 matched the wild type. Sequences with green shading correspond to the 20 bp guide RNA segments employed for the Cas9-mediated knockout, while the sequences with red shading define the adjacent PAM regions, critical for Cas9 binding. Text presented vertically along the left edge outside the rectangles denotes the chromosome numbers of potential off-target sites. Within these rectangles, the HM-1 sequences represent the wild-type reference at these potential off-target sites, with deviations from the respective guide RNA sequence accentuated by red text. The chromatographic peaks shown in different colors correspond to the sequencing readouts for lines 377 and 397, with blue shading underscoring the potential off-target areas. Comparative analysis revealed no doublet peaks in the chromatograms, and the deciphered sequences aligned with the HM-1 reference at the respective sites. The editing using CRISPR-Cas9 in cotton demonstrated high specificity, with no off-target effects detected. (**D**) Scanning electron microscopy images of mature fibers at different magnifications for the wild type (HM-1) and *GhMYB52 Like* mutant (represented by line 377). Scale bar: 30 μm. (**E**) Statistical analysis of manually combed fiber lengths, visualized using violin plots with internal box plots. The blue, red, and green outlines indicate the data density distributions for the wild type (HM-1) and transgenic lines 377 and 397, respectively. The internal box plots range from the 10th to the 90th percentiles, covering the majority of the data. The box boundaries represent the lower and upper quartiles, and the median is indicated by the central line. Black dots signify outliers beyond the 10th to 90th percentile range. “ns” denotes non-significance, while “***” indictes high statistical significance, with a *p*-value less than 0.001. Each group corresponds to 60 measurements. (**F**) Morphology of combed cotton seeds from each line. Scale bar: 10 mm.

To investigate the regulatory role of GhMYB52 Like in the biosynthesis of the SCW in cotton fiber cells, we utilized gene-editing technology (CRISPR-Cas9) to induce random mutations in the coding sequences of *Gh_A12G2460* and *Gh_D12G2588*. Two CRISPR-Cas9 target sites were designed within the exons of *Gh_A12G2460* and *Gh_D12G2588* (Figure 3A), resulting in the generation of 24 transgenic cotton seedlings via *Agrobacterium*-mediated transformation. Sanger sequencing identified two transgenic cotton plants in the T_0_ generation with biallelic mutations in the A and D subgenomes, respectively, namely, lines 377 and 397. Through screening and propagation in the T_1_ generation, a sufficient quantity of cotton plants with homozygous mutations in both the A and D subgenomes, respectively, was created by the T_2_ generation. Figure 3B schematically depicts the edited coding region sequences of lines 377 and 399 in the T_2_ generation, where deletions in the D subgenome of both lines lead to a frameshift resulting in the premature termination of translation. In line 377, a base deletion at site 1 in the A subgenome caused immediate translation termination; in line 399, a substantial deletion between sites 1 and 2 in the A subgenome resulted in the loss of a critical 129 amino acids within the binding domain. These findings demonstrate that the edits effectively abolished GhMYB52 Like function in both lines. The designed sgRNA sequences exhibit high specificity for targeting *GhMYB52 Like*, without affecting other members of the MYB family. To further assess the off-target effects of CRISPR-Cas9 in lines 377 and 397, sequencing analysis was conducted on the two most likely off-target sites for each target sequence, encompassing a genome-wide assessment of several plants. As shown in Figure 3C, all mutations identified at potential off-target sites matched the control HM-1 sequence, demonstrating gene editing’s high specificity and the absence of off-target effects.

Since *GhMYB52 Like* was specifically expressed during fiber SCW development, it may play an important role in determining fiber yield or fiber quality. We first compared the changes in the mature fiber yield traits between wild-type and T_2_ generation materials. Yield trait data were robustly supported by an adequate number of T_2_ generation materials. As demonstrated in Table 1, significant decreases in yield traits were observed in lines 377 and 397 compared to the wild type. These included reductions in boll weight (by −6.48% for line 377 and by −11.22% for line 397), single-boll fiber weight (by −9.04% for line 377 and by −11.86% for line 397), and lint index (by −5.74% for line 377 and by −7.08% for line 397) compared to the wild type (HM-1). Additionally, line 397 exhibited decreases in the number of seeds per boll (by −6.69%) and the seed index (by −6.42%). However, the functional knockout of *GhMYB52 Like* in cotton did not significantly affect the fiber quality parameters, including fiber length, fiber strength, and micromaire value (Table 2 and Figure 3F). Electron microscopy imaging of mature fibers (Figure 3D) indicated that there were no significant differences in the twist or surface texture of the fibers in line 377. 

### 2.4. GhMYB52 Like Does Not Directly Regulate the Transcription of Cellulose Synthase-Related Genes

**Figure 4:**
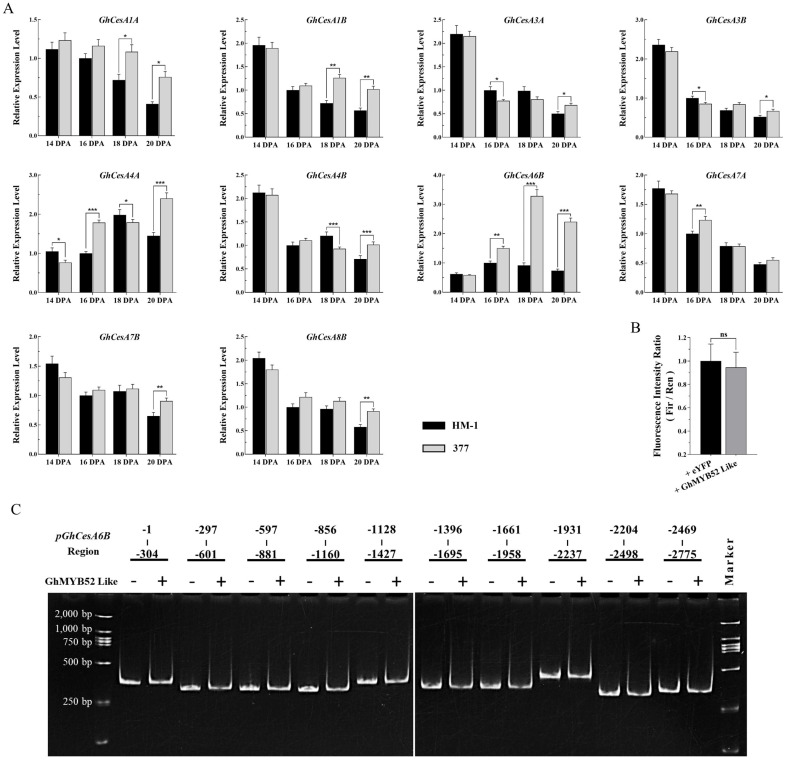
Relative expression of cellulose synthase gene family members and validation of GhMYB52 Like as a regulatory factor. (**A**) Analysis of relative expression levels of cellulose synthase gene family members. This qRT-PCR analysis investigated key cellulose synthase genes essential for cellulose biosynthesis throughout cotton fiber development. It focused on the homologs of CesA1/3/6, associated with primary cell wall development, and CesA4/7/8, implicated in SCW development, in cotton. This study encompasses fiber cells collected at four developmental stages—14, 16, 18, and 20 DPA—from both the wild type (HM-1) and the *GhMYB52 Like* mutant (line 377). *GhHistone3* was used as the internal reference gene, and each experiment was repeated at least three times. Asterisks indicate levels of statistical significance, where * denotes *p* < 0.05, ** denotes *p* < 0.01, and *** denotes *p* < 0.001. (**B**) Dual-luciferase reporter assay was utilized to investigate the potential regulatory effect of GhMYB52 Like on the *GhCesA6B* promoter. A 3025 bp sequence upstream of the *GhCesA6B* gene’s (*Gh_D05G2313*) start codon was inserted into the pGreenII-0800 vector to act as the reporter. GhMYB52 Like driven by the CaMV35S promoter served as the test effector, while eYFP driven by the CaMV35S promoter functioned as the control effector. To assess regulatory effects, transient expression analyses were conducted on tobacco leaf cells, comparing the intensity ratios of Firefly to Renilla Luciferase signals. Compared to the eYFP control group, GhMYB52 Like did not demonstrate the ability to regulate the *GhCesA6B* promoter. “ns” indicates non-significance. (**C**) Results of electrophoretic mobility shift assay (EMSA). PCR was utilized to create a series of consecutively truncated fragments upstream of the *GhCesA6B* translation initiation site, which were subsequently incubated with GhMYB52 Like protein. Gel electrophoresis and ethidium bromide staining were performed to identify DNA–protein interactions, using treatments devoid of protein as controls. In the illustration, horizontal lines divide different promoter fragment groups, with the numerals above delineating each fragment’s relative position with respect to *GhCesA6B* (the translation start site is designated as zero, upstream promoter regions are marked with negative values, and higher absolute values reflect increased distances from the start site). The symbols “+” and “−” beneath the horizontal lines denote the addition of GhMYB52 Like protein and its absence, respectively.

Considering the direct influence of cellulose accumulation on fiber yield, we investigated the transcription levels of genes associated with cellulose synthase in fiber cells at 14, 16, 18, and 20 DPA to understand the factors responsible for the reduced lint production observed. *GhCesA4B*, *GhCesA7B*, and *GhCesA8B*, which play major roles during the secondary growth stage of cotton fiber cells [21], did not show significant changes in transcription levels (Figure 4A). Notably, within the mutant fibers at 16, 18, and 20 DPA, the transcription levels of *GhCesA1A*, *GhCesA1B*, *GhCesA3A*, *GhCesA3B*, *GhCesA4A*, and *GhCesA7A* remained largely unchanged, with the exception of an evident increase in *GhCesA6B* expression (Figure 4A).

To confirm whether GhMYB52 Like downregulates the transcription of *GhCesA6B*, a 3025 bp sequence upstream of the start codon of the *GhCesA6B* gene (*Gh_D05G2313*) was cloned and used in dual-luciferase reporter assays and simplified Electrophoretic Mobility Shift Assays (EMSAs). The luminescence signals from the dual-luciferase assays indicated that overexpression of GhMYB52 Like did not lead to significant changes in the transcriptional activity of the *GhCesA6B* promoter (Figure 4B). Additionally, electrophoretic analysis of DNA from ten different regions of the *GhCesA6B* promoter, following the addition of GhMYB52 Like protein, failed to detect any band shifts that would indicate the binding of the protein to DNA (Figure 4C). These outcomes suggest that GhMYB52 Like does not directly engage in the transcriptional regulation of *GhCesA6B.*

### 2.5. GhMYB52 Like Negatively Regulates Lignin Biosynthesis

**Figure 5:**
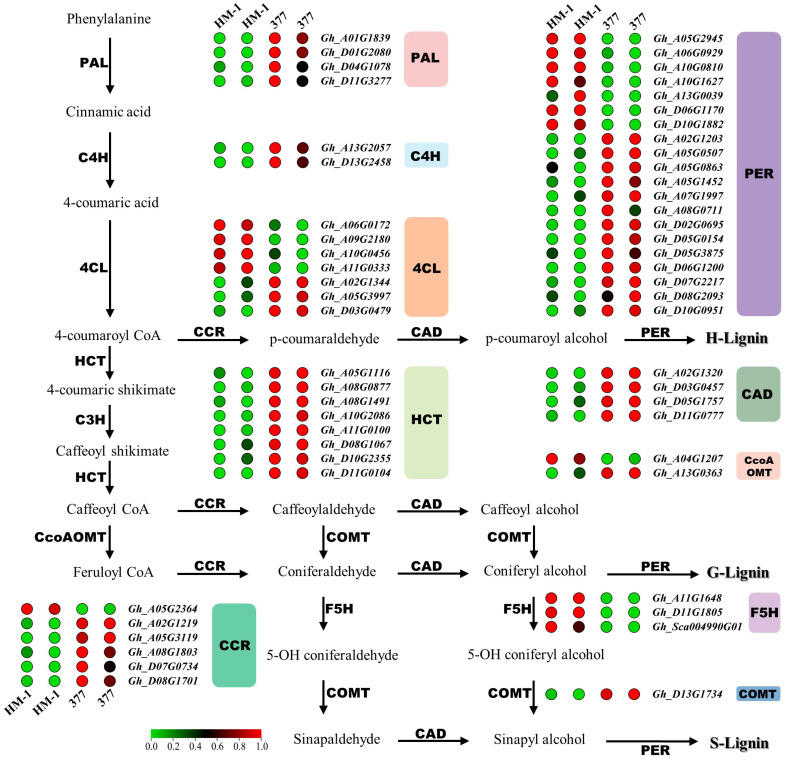
Differential gene expression in the lignin biosynthesis pathway of *GhMYB52 Like*-knockout cotton. This figure illustrates the lignin biosynthesis pathway, with arrows connecting corresponding substrates and products; the abbreviated letters along the arrows represent the enzymes involved in the process. This figure also integrates differentially expressed genes (denoted by colored dots and gene IDs) obtained from the transcriptional profiling data of 16 DPA cotton fiber samples from both the wild type (HM-1) and the *GhMYB52 Like* mutant (line 377). For robustness, each sample was analyzed using two replicates. Gene expression differences were identified with strict parameters, namely, a minimum count threshold of 5 and an absolute log2 fold change (|log2FC|) of 1 or more, pinpointing the most significantly altered genes in the pathway. The corresponding genes from HM-1 and line 377 are depicted using heatmap dots to illustrate relative transcription levels: within the respective samples, red dots signify higher transcription levels of the gene, while green dots indicate lower expression. This color-coded map facilitates the identification of upregulated and downregulated genes within the pathway, providing insights into the transcriptional reprogramming that occurs in response to the *GhMYB52 Like* mutation. The full names of the enzymes involved in the pathway are as follows: PAL (Phenylalanine Ammonia-Lyase), C4H (Cinnamic Acid 4-Hydroxylase), 4CL (4-Coumarate-Coenzyme A Ligase), HCT (Hydroxycinnamoyl Transferase), C3H (p-Coumarate 3-Hydroxylase), CcoAOMT (Caffeoyl Coenzyme A 3-O-Methyltransferase), CCR (Cinnamoyl-CoA Reductase), F5H (Ferulate 5-Hydroxylase), COMT (Caffeic Acid O-Methyltransferase), CAD (Cinnamyl Alcohol Dehydrogenase), and PER (Peroxidase). In the pathway diagram presented, the conversion of phenylalanine to 4-coumaroyl CoA—encompassing the initial three steps of lignin synthesis involving PALs, C4Hs, and 4CLs—constitutes a core set of reactions shared by both the phenylpropanoid pathway and the phenylpropanoid–acetate pathway. 4-Coumaroyl CoA can then be converted into lignin monomers through subsequent steps mediated by HCTs or CCRs, or it can undergo derivative transformations via enzymes associated with the phenylpropanoid-acetate pathway. It is commonly understood that in angiosperms, the content of S- and G- lignin monomers is greater than that of H-monomers.

To elucidate possible metabolic pathways regulated by GhMYB52 Like, we performed transcriptome sequencing on 16 DPA fibers. The high-throughput sequencing results obtained demonstrated that the knockout of *GhMYB52 Like* induced substantial alterations in the gene expression profile of line 377, affecting 5821 genes, with 3770 genes being upregulated, while 2051 were downregulated. Gene Ontology (GO) enrichment analysis revealed a pronounced enrichment of differentially expressed genes in categories pertinent to cell wall formation, including secondary cell wall biogenesis involved in seed trichome differentiation, plant-type secondary cell wall biogenesis, and xylan metabolic process (Figure S6A). Parallelly, Kyoto Encyclopedia of Genes and Genomes (KEGG) enrichment analysis indicated that GhMYB52 Like may play a pivotal role in pathways related to flavonoid biosynthesis, phenylpropanoid biosynthesis, and cutin, suberine, and wax biosynthesis (Figure S6B). The transcriptomic results support the notion of *GhMYB52 Like*’s participation in the synthesis of the SCW. Moreover, prior investigations suggested MYB52’s potential involvement in regulating lignin biosynthesis [15,17,18,19]. In light of these insights, we delved into the differential gene expression within the phenylpropanoid metabolic pathway. As expected, most differentially expressed genes involved in lignin biosynthesis exhibit an upregulation trend in *GhMYB52* knockout mutants (Figure 5). These data imply that GhMYB52 Like may act as a negative regulator of lignin biosynthesis.

**Figure 6:**
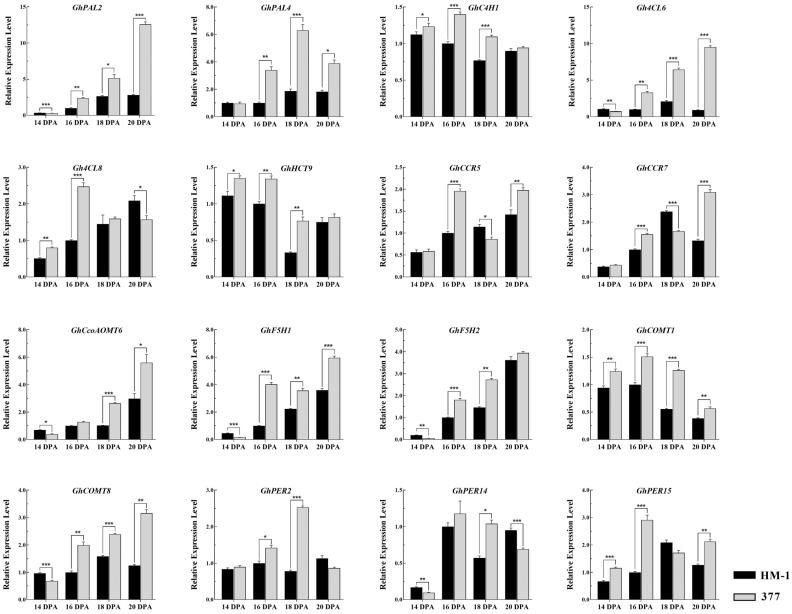
Relative expression analysis of lignin-biosynthesis-related genes. This figure delineates the expression patterns of crucial genes involved in the lignin biosynthesis pathway during the secondary cell wall accumulation phase of cotton fiber cells. Samples were collected from the wild-type HM-1 and the *GhMYB52 Like* mutant line 377 at critical developmental stages—14, 16, 18, and 20 DPA—to monitor the sequential changes in gene expression related to lignin production. The enzymes encoded by the genes under investigation play pivotal roles in the conversion of phenylalanine to lignin monomers through a series of enzymatic reactions. The full names of the enzymes involved are as follows: PAL (Phenylalanine Ammonia-Lyase), C4H (Cinnamic Acid 4-Hydroxylase), 4CL (4-Coumarate-Coenzyme A Ligase), HCT (Hydroxycinnamoyl Transferase), CcoAOMT (Caffeoyl Coenzyme A 3-O-Methyltransferase), CCR (Cinnamoyl-CoA Reductase), F5H (Ferulate 5-Hydroxylase), COMT (Caffeic Acid O-Methyltransferase), and PER (Peroxidase). Among the enzymes analyzed, PALs, C4Hs, and 4CLs play roles in common steps central to both the phenylpropanoid and phenylpropanoid-acetate pathways. Subsequent enzymatic reactions catalyze the formation of distinct lignin monomers. For a detailed metabolic pathway map, please refer to Figure 5 and its corresponding annotation. *GhHistone3* was used as the internal reference gene, and each experiment was repeated at least three times. Asterisks indicate levels of statistical significance, with * denoting *p* < 0.05, ** denoting *p* < 0.01, and *** denoting *p* < 0.001.

To confirm the transcriptome-sequencing findings, transcriptional levels of a set of genes that are pivotal to SCW development were detected. Significant upregulation of *GhPAL2*, *GhPAL4*, *GhC4H1*, *Gh4CL6*, *GhHCT9*, *GhCcoAOMT6*, *GhF5H1*, *GhF5H2*, *GhCOMT1*, and *GhCOMT8* across different stages of secondary fiber development was found (Figure 6). Although *Gh4CL8*, *GhCCR5*, *GhCCR7*, *GhPER2*, *GhPER14*, and *GhPER15* exhibited transcriptional downregulation at certain time points, their overall expression trended upwards during the secondary growth phase (Figure 6). These qRT-PCR data validated the increase in the transcription levels of lignin-biosynthesis-related genes consequent to the knockout of *GhMYB52 Like*. In conclusion, the disruption of GhMYB52 Like elicited the upregulation of a specific gene set related to lignin biosynthesis, highlighting its negatively regulatory function in the lignin deposition process in cotton fibers.

**Figure 7:**
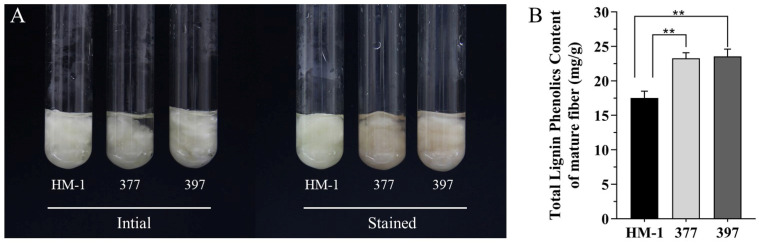
Coloring and quantification of lignin in mature cotton fibers. (**A**) Total lignin visualization in mature fibers via phloroglucinol staining. This image provides a visualization of the lignin content within mature cotton fibers from three different upland cotton lines: HM-1, 377, and 397. The term “Initial” refers to fibers prior to staining, exhibiting their natural, untreated state. The term “Stained” delineates the alteration in hue following a 45 min phloroglucinol staining regimen. This regimen resulted in a reddish-brown coloration in lines 377 and 397, signifying an augmentation of lignin deposition. (**B**) Total lignin content measurement in mature fibers. The acetyl bromide method was applied to intact fiber samples to ascertain the total lignin concentration. The bar graph conveys the quantified lignin content in the mature fibers of the three lines: HM-1, 377, and 397. Alkali lignin was employed as a reference for generating a standard curve, ensuring accurate lignin quantification. The measured lignin content is expressed in milligrams per gram of mature fiber, with the data points averaging the results of multiple measurements for each line. Error bars indicate standard deviations, verifying the repeatability and reliability of the results. The double asterisks (**) above the bars signify a statistically significant difference in lignin content between the varieties, with the level of significance set at *p* < 0.01. The lines 397 and 377 are highlighted as having a noticeably higher lignin content compared to that of HM-1.

To further confirm GhMYB52 Like’s role in lignin biosynthesis, we detected lignin content via the phloroglucinol staining of the mature fibers. A visible reddish-brown color was observed in the fibers of lines 377 and 397, indicating the evident increase in lignin deposition in these fibers (Figure 7A). Quantitative analysis conducted using the acetyl bromide method showed that total lignin content increased to 23.29 ± 0.81 (mg/g) and 23.56 ± 1.05 (mg/g) in lines 377 and 397, respectively, from 17.55 ± 0.97 (mg/g) in HM-1, marking increases of 32.73% and 34.26%, respectively, in mature fibers. These findings corroborate that GhMYB52 Like functions as a negative regulator in the lignin biosynthetic pathway.

## 3. Discussion

### 3.1. The Functional Knockout of GhMYB52 Like Does Not Directly Regulate Cellulose Deposition in Cotton Fiber Cells

Members of the R2R3 MYB transcription factor family are abundant in different plant species, though the exact number varies from one species to another [6,22,23,24]. Our findings reveal that R2R3 MYBs constitute the largest segment within the MYB superfamily in cotton, accounting for 414 out of the 733 members. Furthermore, R2R3 MYB members demonstrate distinct preferential expression patterns across diverse cotton tissues (Figure 1), indicating this gene family’s integral role in various phases of cotton development and growth. The composition of the secondary cell wall (SCW) is predominantly cellulose, hemicellulose, and lignin. The deposition of the SCW is vital for determining the quality of cotton fibers. Since the transcription and translation of transcription factors occur prior to the regulation of their downstream target genes, and as transcriptional regulation related to the deposition of cotton fiber SCWs is most active between 15 and 25 DPA, the period in which these processes take place is critical for fiber development. Among the 26 candidate R2R3 MYB genes identified to play roles in SCW development in cotton (Table S2), *Gh_D13G2261* and the *MYB52* homologs *Gh_D12G258*8 and *Gh_A12G2460* show highly specific expression at 20 DPA. This suggests these three genes have a significant impact on the synthesis of fiber SCW. Additionally, there is evidence that MYB52 participates in SCW synthesis, although its precise function remains to be fully clarified. Transcript-level analyses of wild-type cotton have also shown that *GhMYB52 Like* has more specific expression in fibers compared to *GhMYB52*, indicating a significant role in the synthesis of the secondary cell wall in fiber cells. Consequently, this study focuses primarily on *GhMYB52 Like*.

Transcriptional regulation emerges as a pivotal mechanism in the biosynthesis of the SCW, a process believed to be governed by a hierarchical structure with at least three levels of transcriptional control [9,25,26,27]. AtSND1 has been identified as a primary regulatory switch in SCW synthesis, with research conducted by Zhong et al. illustrating AtMYB52’s position downstream of AtSND1. Experiments involving AtMYB52 Dominant Repression have demonstrated a notable decrease in the thickening of SCWs, positioning AtMYB52 as a positive regulator in *Arabidopsis*’ SCW deposition [28]. AtMYB46 and AtMYB83 exhibit functional redundancy and serve as direct targets of AtSND1 [16,26]. Their roles as secondary switch factors in SCW synthesis are underscored by the overexpression of AtMYB46 or AtMYB83, leading to the accumulation of cellulose, xylan, and lignin, with AtMYB52 being positively regulated by both [16,29]. Transient expression assays conducted on *Arabidopsis* mesophyll cell protoplasts revealed that AtMYB52 and AtMYB54 can activate the transcriptional activities of promoters for cellulose synthase (CesA8), the xylan biosynthesis gene (IRX9), and the lignin synthesis gene (4CL1). However, AtMYB52’s overexpression in transgenic *Arabidopsis* did not manifest in discernible changes in the thickness of SCWs in fibers and vessels [28], suggesting MYB52’s responsiveness to key factors in SCW formation, with no conclusive evidence on its impact on cellulose synthesis.

Cotton fibers originate from single epidermal cells of an ovule, progressing through distinct yet overlapping developmental stages, including fiber initiation, rapid elongation, SCW synthesis, and dehydration-induced maturation [12]. Given that mature cotton fibers contain over 94% cellulose, they represent an exemplary model for investigating cell wall and cellulose biosynthesis [10,11]. Our initial focus was investigating the effects of GhMYB52 Like on cellulose deposition alterations in fiber cells. Scanning electron microscopy analysis of mature fibers did not reveal significant changes, preliminarily suggesting that GhMYB52 Like has a limited impact on cellulose deposition. We also incidentally observed the fiber initiation stage in both wild-type and transgenic materials using scanning electron microscopy, noting no discernible differences between them. The phenomenon is in line with the absence of *GhMYB52 Like* transcription during the initial phase of fiber development. Upon manual measurement of mature fiber lengths (Figure 3E,F), an increase in length was noted for cotton fibers in the 397 line. However, this observation was not supported by the fiber quality assessment outcomes (Table 2). Considering the transcriptional regulation of genes associated with cellulose synthase and lignin synthesis, among other factors (detailed subsequently), we deduced that the functional absence of GhMYB52 Like does not influence the length of mature cotton fibers. The micronaire, which is a value used for assessing the fineness of mature fiber cells and a macroscopic indicator of SCW deposition, remained unchanged in transgenic lines, as shown in Table 2, tentatively indicating that there are no significant alterations in cellulose deposition within cotton fibers.

To further elucidate GhMYB52 Like’s role in modulating cellulose deposition within the SCW, we analyzed the relative expression of ten cellulose synthase gene families at four developmental stages in fiber cells. The influence of these genes on fiber development, as indicated by transcription levels, is depicted in the FPKM heatmap (Figure S7). Although CesA6 is traditionally viewed as a component of the primary cell wall cellulose synthase complex, Carroll et al.‘s findings suggest that there are potential interactions between cellulose synthases of the primary cell wall (CesA1/CesA3/CesA6) and those linked to the secondary cell wall (CesA4/CesA7/CesA8), forming functional complexes [30]. Further in vitro analyses have refuted the likeliness of GhMYB52 Like negatively regulating *GhCesA6B*. Another study on GhMYB52 Like in cotton further supports the conclusion that GhMYB52 Like is not involved in the cellulose biosynthesis of fiber cells [31]. Du’s generation of transgenic cotton with overexpressed and antisense-suppressed *Gh_A12G2460* showed that there were no uniform changes in cellulose concentration or cell wall thickness in T_0_ mature fibers. Therefore, fibers from *GhMYB52 Like* knockout mutants exhibited no significant visible changes.

### 3.2. GhMYB52 Like Negatively Regulates Lignin Biosynthesis in Fiber Cells

Phenolic compounds are primarily synthesized from the frameworks of phenylpropanoids and phenylpropanoid-acetate esters. Lignin, a complex phenylpropanoid polymer that resides between cellulose and hemicellulose within the SCW, significantly enhances the cell wall’s mechanical strength [32]. The genes related to the lignin biosynthesis pathway fall under the meticulous control of the previously outlined multi-layered transcriptional regulatory network specific to the SCW [33]. In *Arabidopsis*, the transcription factors MYB58 and MYB63 are positively regulated by SND1, its closely related homologs, and MYB46, contributing to the activation of lignin biosynthesis in the SCW [34]. There is evidence indicating that AtMYB52 and AtMYB63 markedly upregulate the expression of PAL4, a crucial enzyme initiating lignin biosynthesis, acting as potent activators [15]. The overexpression of MdMYB52 (MD05G1011100, an ortholog of AtMYB52) in tobacco leaves resulted in a significant enhancement of lignin content, underscoring MYB52’s pivotal role in promoting lignin biosynthesis and accumulation [17]. Cassan-Wang et al. observed intensified autofluorescence and phloroglucinol staining in the lignified tissues of an atmyb52 T-DNA insertional mutant compared to the wild type, hinting at MYB52’s potential in regard to dampening lignin accumulation [18]. This stands in contrast to the findings of Zhong et al., who noted a marked decrease in SCW thickness in AtMYB52 Dominant Repression experiments without observing changes in the secondary walls of fibers and vessels in AtMYB52-overexpressing transgenic *Arabidopsis* [28]. Conversely, Chai et al. reported that overexpressing PdMYB90/167 (AtMYB52 orthologues) in *Arabidopsis* led to the thinning of the SCW in vessels and fibers and a decrease in xylose, cellulose, and lignin levels in six-week-old plants [19]. Therefore, the mode of MYB52-mediated regulation of lignin synthesis, whether positive or negative, may vary among different plant tissue types.

Evidence suggests MYB52’s involvement in lignin biosynthesis, though the corresponding regulatory mechanisms and target genes remain to be fully elucidated. Taking into consideration that cotton fibers are uniform single cells, our findings demonstrate that GhMYB52 Like does not affect the biosynthesis of cellulose in the SCW, establishing cotton fiber cells as an ideal model for the further exploration of GhMYB52 Like’s role in lignin biosynthesis with minimal interference from other secondary wall components. Furthermore, in typical tissue types, lignin metabolic byproducts, such as ferulic, caffeic, and coumaric acids, contribute phenolics to suberin. The negligible suberin levels in white cotton fibers render them particularly conducive to lignin biosynthesis research, as they are not affected by suberin synthesis [35]. Our conclusions regarding GhMYB52 Like’s negative modulation of lignin biosynthesis align with the observations of Cassan-Wang et al. [18] and the outcomes obtained by Chai et al. [19]. The expression analysis depicted in Figure 6 reveals no clear trend of gene upregulation in fibers at 14 DPA, potentially due to *GhMYB52 Like* initiating transcription at 13 DPA. This delay indicates that GhMYB52 Like may not act independently in repressing lignin biosynthesis genes, implying a potential requirement for additional regulatory factors. In the lignin biosynthesis pathway, the initial three steps involving PAL, C4H, and 4CL constitute the common core of both the phenylpropanoid and phenylpropanoid-acetate pathways. Consequently, *Gh4CL8*’s expression may also be subject to joint regulation by transcription factors associated with the phenylpropanoid-acetate pathway, leading to inconsistent transcription levels on different days. Although bioinformatics predictions have identified a plethora of peroxidase activity proteins in cotton, Figure S8 still lists a significant number of proposed *GhPER* family members, indicating the need for further physiological and biochemical evidence to determine which are specific to the phenylpropanoid pathway. Notably, the qRT-PCR results for *GhF5H1* and *GhF5H2* contradict those shown in the transcriptome, and we place more confidence in the outcomes obtained using specific primers. The cotton genome harbors a limited number of ferulate 5-hydroxylase gene members, among which *GhF5H1* is predominantly expressed in cotton fibers. The negative regulation of this gene by GhMYB52 Like could better explain the observed increase in lignin content.

Upon reviewing the currently published hierarchical gene regulatory network for SCW synthesis, *GhMYB52 Like* is positioned at the third level, directly regulating the transcription of downstream functional genes. Our hypothesis posits that GhMYB52 Like associates with a specific sequence, inferred from the findings presented in Section 2.4 and Section 2.5, likely located within the promoter regions of genes involved in lignin biosynthesis as opposed to those associated with cellulose synthase. To explore this hypothesis, we conducted an analysis of 52 promoter sequences, each extending 2.5kb upstream from the translation initiation sites of 26 gene pairs, utilizing six potential DNA matrix sequences recognized by AtMYB52. Table S3 summarizes the presence of various cis-acting elements within these promoters, and statistical analysis revealed that none of these six elements exhibited the anticipated pattern. This discrepancy indicates that the GhMYB52 Like binding sequence in cotton markedly differs from the AtMYB52 binding sequence known in *Arabidopsis*. Prior studies have highlighted the AC element’s prevalence in the promoter regions of lignin biosynthesis genes [36], with both activators and repressors being capable of binding to it [37]. Furthermore, sites such as SMRE and M46RE are posited to be potential loci for lignin biosynthesis gene regulation [38,39]. Whether GhMYB52 Like binds to these known cis-acting elements or possesses unique binding sites requires further experimental validation. 

The systematic changes brought about by the functional knockout of *GhMYB52 Like* in cotton yield traits are discussed herein. Cotton boll weight is influenced by the aggregate weight of fibers and seeds. Compared to the wild-type plants, cotton with the *GhMYB52 Like* gene knocked out exhibited no significant changes in Boll Seed Count or Seed Index, implying that the significant reduction in boll weight was primarily due to decreased fiber mass. The observed decrease in the lint index is typically ascribed to either diminished initial fiber counts or reduced cellulose deposition. Upon reviewing existing data, it is evident that *GhMYB52 Like* neither disrupts cellulose synthesis nor impacts the onset of fiber development. Considering the identified role of *GhMYB52 Like* in negatively regulating lignin biosynthesis and noting that mature white cotton fibers predominantly consist of cellulose with minimal amounts of other substances, it is logical to infer that the accumulation of lignin within fiber cells contributes to the decline in the lint index. This proposition is based on a thorough evaluation of the present findings and necessitates additional research for confirmation. Naturally green cotton contains higher levels of suberin, and naturally colored cotton is characterized by lower yields [40]. This seems to be one of the indications that augmenting the phenylpropanoid pathway may lead to a decrease in yield. In summary, the knockout of *GhMYB52 Like* function both leads to the accumulation of lignin in fiber cells and significantly reduces the lint index, a crucial indicator of quality.

In conclusion, unveiling the mechanisms behind the formation of plant secondary walls holds considerable significance for plant biology. While the biosynthesis pathways of secondary walls have been elucidated to some extent, the intricacies of the transcriptional regulatory network have yet to be fully comprehended. Given that lignin is a primary constituent of secondary walls, investigating its transcriptional regulation is pivotal for grasping the molecular underpinnings of SCW development. This investigation has spotlighted an R2R3 MYB transcription factor, GhMYB52 Like, that exhibits preferential expression during the secondary wall developmental phase in fiber cells. Through targeted gene knockout in *Gossypium hirsutum*, we have ascertained that GhMYB52 Like plays a role in downregulating lignin biosynthesis without affecting cellulose biosynthesis. Despite the absence of direct evidence pinpointing the exact genes related to lignin biosynthesis that GhMYB52 Like modulates, the findings sufficiently demonstrate GhMYB52 Like’s capacity to repress lignin biosynthesis and delineate a set of potential target genes. Additionally, *GhMYB52 Like* was found to have a positive impact on the lint index of cotton. In accordance with these insights, we suggest that lignin accumulation in fiber cells plays a key role in the observed reduction in the cotton lint index. Such insights are not only crucial for understanding cotton fiber cell development but also hold significance for harnessing secondary wall biosynthesis in the creation of innovative plant-based materials.

## 4. Materials and Methods

### 4.1. Plant Materials and Plant CRISPR-Cas9 Gene-Editing Vector

In this study, a wild-type upland cotton (*Gossypium hirsutum* L.) HM-1 cultivar was used. Transgenic cotton seedlings were obtained using the tissue culture techniques described by Luo et al. [41]. All plants were grown at Southwest University (Chongqing, China), with field experiments taking place from May to October. Homozygous knockout mutants were employed for tissue material collection and data analysis.

All vectors constructed in this study were generated using the seamless cloning method (C112/C113, Vazyme, Nanjing, China). The dual-expression vector for cotton gene editing, pRGEB32-GhU6.7-NPTII, was kindly provided by Professor Shuang-Xia Jin of Huazhong Agricultural University [42]. The specific Guide RNA sequences were designed using the online tool CRISPR-P, which also provided information on potential off-target sites [43]. The construction of a vector targeting multiple knockout sites was carried out based on the methodology published by Xie et al. [44].

### 4.2. Bioinformatics Analysis

The reference genome information for upland cotton (*Gossypium hirsutum*, NAU-NBI, v1.1) along with the gene expression data for the upland cotton TM-1 cultivar (Fragments Per Kilobase per Million, FPKM) were both obtained from the Key Laboratory of Crop Genetics and Germplasm Enhancement, Cotton Hybrid R&D Engineering Center at Nanjing Agricultural University [45]. Genome-wide domain scan was performed using InterProScan (version 5.62-94.0) in a Linux environment, utilizing the Pfam database (version Pfam-35.0). This analysis focused on the R2R3 MYB family, which was identified by the presence of two conserved and non-overlapping Myb-like DNA-binding domains (Pfam accession number: PF00249) within the structures of the members of this family.

Collinearity relationships and expression heatmaps were analyzed and visualized using Tbtools-II [46]. Protein 3-Dimensional structure predictions were performed using AlphaFold2 (advanced v2) [47]. Phylogenetic trees were constructed using MEGA software (version 11.0.13) via the Neighbor-Joining method, with 1000 bootstrap replicates, and visualized using the online tool Chiplot [48]. Statistical analyses, including bar graphs and biostatistical *t*-tests, were executed using GraphPad Prism software (version 8.0.2), with results presented as means ± standard deviation.

### 4.3. Screening and Validation of Knockout Lines

Homozygously edited transgenic plants were screened using PCR and Sanger sequencing, with off-target site detection performed subsequently. Specific primers, capable of distinguishing between the A/D subgenomes, were designed to amplify sequences surrounding the editing sites for sequencing. The DSDecodeM online tool facilitated the interpretation of overlapping peaks in chromatograms from multiple sequences [49]. In the T_2_ generation, ten plants from cotton lines 377 and 397 were randomly selected, and sequences near the two most probable potential off-target sites for each target sequence were amplified and sequenced for assessment.

### 4.4. Quantitative Real-Time PCR (qRT-PCR) Analysis

Field-fresh tissue samples were collected, ground in liquid nitrogen, and used for total RNA extraction using the EASY Spin Plant RNA Rapid Extraction Kit (RN09, Aidlab, Beijing, China). A total of 1 μg of total RNA was used for genomic DNA removal and first-strand cDNA synthesis using the HiScript II Q RT SuperMix for qRT-PCR Kit (R223, Vazyme). The qRT-PCR reaction mixture (20 μL) included 10 μL of Universal SYBR qPCR Master Mix (Q711, Vazyme), 5 μL of diluted cDNA, 0.5 μL of 10 μmol/L forward and reverse primers each, and 4 μL of ddH_2_O. Reactions were induced in triplicate, with fluorescence detection enabled via a Bio-Rad CFX Connect Real-Time PCR System, using the ΔΔCq method. *GhHistone3* (Accession number: AF024716) served as the reference gene, with specific primer sequences provided in the Supplementary Materials.

### 4.5. Subcellular Localization Observation

Subcellular localization signals of the target protein were observed via transient expression in tobacco leaf epidermal cells [50]. The coding sequence of *GhMYB52 Like* (*Gh_D12G2588*) from the D subgenome, excluding the stop codon, was cloned into the pCambia-*cEGFP* vector for expression of the GhMYB52 Like::eGFP fusion protein. This recombinant vector was transformed into the GV3101 (*Agrobacterium tumefaciens*) strain using standard electroporation. Cultured *Agrobacterium*, harvested in the logarithmic growth phase, was resuspended in a solution [50 mM MES, pH 5.6; 0.5% (*w*/*v*) D-glucose; 2 mM Na_3_PO_4_; 100 μM acetosyringone] and injected into the dorsal side of the leaves of 4-week-old *Nicotiana benthamiana* plants using a 1 mL syringe. After 2–3 days of growth, leaf sections were stained with DAPI and observed under a laser confocal microscope (SP8, Leica, Wetzlar, Germany).

### 4.6. Transcriptional Activation Experiments of GhMYB52 Like

The transcriptional activation potential of GhMYB52 Like was evaluated using the yeast two-hybrid system. In the context of the reporting system, the G1 promoter in the Y2H strains activates the *HIS3* gene, while the M1 promoter activates both the *AUR1-C* and *MEL1* genes. HIS3 is capable of conferring resistance to histidine deficiency, AUR1-C aids in conferring resistance to the selection pressure of AbA, and α-galactosidase (MEL1) facilitates the process by which the chromogenic substrate X-alpha-Gal turns blue.

Full-length, N-terminal, and C-terminal sequences of GhMYB52 Like (*Gh_D12G2588*) were cloned into the bait vector pGBKT7. These vectors were then transformed into the yeast strain Y2H (*Saccharomyces cerevisiae*). Monoclonal transformants were cultured in tryptophan-deficient liquid medium. Serial dilutions of these cultures were spotted onto solid media lacking histidine/tryptophan, supplemented with 200 ng/mL Aureobasidin A (AbA) and 40 μg/mL X-alpha-Gal, to conduct spot assays. Spot assays on media lacking tryptophan served as the growth control.

### 4.7. Statistical Analysis of Cotton Yield Traits and Fiber Quality Assessment

The materials used for the statistical analysis of yield traits were all obtained during the same harvest time point. The data presented in the statistical tables were calculated as follows: Boll weight is the weight of the seed cotton divided by the number of bolls. Boll seed count is calculated by dividing the total number of seeds by the number of bolls. Lint percentage is determined by dividing the weight of lint cotton by the weight of seed cotton. Single boll fiber weight is obtained by multiplying the weight of a single boll by the lint percentage. The seed index is measured by weighing 100 seeds with nine replicates for each sample. Finally, the lint index = (seed index × lint percentage)/(1 − lint percentage). Each set of data, with the exception of the seed index and lint index—which each have nine replicates—is underpinned by at least 60 cotton bolls; these were randomly divided into three groups.

The fiber quality analysis was performed by Supervision Inspection and Test Center of Cotton Quality, Ministry of Agriculture and Rural Affairs of China. Mature seed cotton was harvested from both knockout and control plant lines on the same day, followed by drying, ginning, and cleaning before submission for testing. The physical properties of the lint cotton, including upper-half mean length, uniformity index, breaking tenacity, micronaire value, and elongation rate, were quantitatively assessed using a high-capacity integrated cotton fiber tester (model HVI-1000), adhering to the GB/T 20392-2006 standards [51]. Three samples of each material were prepared for evaluation.

The determination of hand-combed fiber length was carried out on seed cotton harvested from the same batch, measuring the length of 60 neatly combed upper halves for statistical analysis.

### 4.8. Observation under a Scanning Electron Microscope (SEM)

For the observation of the initial stages of fiber cells, cotton bolls at 0 DPA, 1 DPA, and 2 DPA were collected post 10:00 am on the day of observation. Ovules from the same portion of the cotton bolls were prepared, frozen in liquid nitrogen, and then observed under an S-3400N (Hitachi, Tokyo, Japan) scanning electron microscope.

For mature fiber observation, seed cotton was collected, and fibers were uniformly cut, laid flat, and adhered to the sample stage for SEM observation using an S-3400N scanning electron microscope.

### 4.9. Dual-Luciferase Reporter Assay

The 3025 bp sequence upstream of the start codon of the *GhCesA6B* gene (*Gh_D05G2313*) was cloned into the pGreenII 0800 vector to create the Reporter construct (pGreenII 0800-*pGhCesA6B*). A construct with the CaMV35s promoter driving the expression of GhMYB52 Like in the pCambia-*GhMYB52 Like* vector acted as the test effector. The pCambia-*eYFP* vector, under the control of the CaMV35s promoter for eYFP expression, served as the control effector. These vectors were transformed into the *Agrobacterium tumefaciens* strain GV3101 via electroporation, with the transformation of pGreenII 0800-*pGhCesA6B* necessitating the helper plasmid pSoup [52].

Following the previously described procedure, bacterial suspensions prepared for inoculation were mixed in a 1:1 (*v*/*v*) ratio of the respective effector-to-reporter cultures. After the inoculation of tobacco leaves with the mixed bacterial suspension, the plants were grown at 22–25 °C for 60 h. Leaf discs were punched out and immediately frozen in liquid nitrogen. At least 3 replicates were set up for each sample. The discs were then ground with a small pestle, and extracts were prepared in potassium phosphate buffer (100 mM, pH 7.8) containing 1mM DTT. After centrifugation at 4 °C and 12,000 rpm for 10 min, the supernatant was collected for testing. The luciferase signals were detected according to the Dual-Glo Luciferase Assay System (E2920, Promega, Madison, WI, USA) protocol. Data analysis was performed based on the ratio of Firefly to Renilla Luciferase signal intensities.

### 4.10. Electrophoretic Mobility Shift Assay (EMSA)

The *Escherichia coli* NusA protein (Accession number: NP_417638.1), serving as a solubility-enhancing factor, had its coding sequence fused upstream of the *GhMYB52 Like* (*Gh_D12G2588*) coding sequence. The fusion gene was cloned into the pCold-I vector. The recombinant pCold-*NusA-GhMYB52 Like* plasmid was transformed into the protein expression strain OrigamiB (DE3) for the expression of the fusion protein. The expression strain was cultured in 2 × YT medium containing ampicillin until the logarithmic growth phase, induced with a final concentration of 0.3 mM IPTG, and incubated at 15 °C, 200 rpm for 24 h. After induction, the cells were collected via centrifugation, resuspended in PBS, lysed via sonication, and purified using Ni magnetic beads (V8500, Promega). The eluted protein was desalted using ultrafiltration tubes, supplemented with a final concentration of 30% glycerol, aliquoted, and stored at −80 °C.

Specific primers were designed to amplify and subsequently gel-purify probe sequences using the pGreenII 0800-*pGhCesA6B* plasmid as a template. The binding conditions between the probe and GhMYB52 Like protein were established in accordance with the LightShift Chemiluminescent EMSA Kit (20148, Thermo, Waltham, MA, USA) guidelines. The binding reaction mixture, totaling 20 μL, contained 150 ng of the probe and 8 μL of protein and was incubated at room temperature for 45 min before being loaded onto a PAGE gel for electrophoretic separation. Electrophoresis conditions were also set, following the kit’s manual. After electrophoresis, the PAGE gel was stained directly with ethidium bromide (EB) and imaged using a UV gel documentation system.

### 4.11. Transcriptome Analysis

Cotton bolls from the HM-1 and 377 lines at 16 DPA were collected, and the fiber was rapidly frozen in liquid nitrogen before storage at −80 °C. Samples were sent to Majorbio Bio-Pharm Technology Co., Ltd. (Shanghai, China) for RNA extraction and high-throughput sequencing, with two replicates for each sample. Data analysis was performed on an online platform facilitated by Majorbio, utilizing FPKM (fragments per kilobase of transcript per million mapped reads) as the metric for expression levels. For differential expression analysis, DESeq2 was employed, applying an adjusted *p*-value threshold of less than 0.05. The fold-change threshold was determined to be either 2 or 10, tailored to the specific aims of this study (for detailed information, please consult the Section 2 and figure captions). The “*Gossypium hirsutum* (AD1) TM-1 genome NAU-NBI_v1.1” served as the reference genome (https://mascotton.njau.edu.cn/info/1054/1118.htm, accessed on 5 May 2015). The raw transcriptome data generated in this study have been deposited in the Sequence Read Archive (SRA) under the accession number “PRJNA1087552”.

### 4.12. Cis-Acting Element Scanning

The six DNA binding motifs of *Arabidopsis* MYB52 were downloaded from footprintDB (https://footprintdb.eead.csic.es/index.php, accessed on 22 December 2023). The accession numbers for these motifs are as follows: MYB52 (*Arabidopsis*PBM 20140210); MYB52_2 (*Arabidopsis*PBM 20140210); AtMYB52_I (AthaMYB 1.0); AtMYB52_II (AthaMYB 1.0); MA1171.1 (JASPAR 2024); and MA1171.2 (JASPAR 2024). Promoter sequences for the genes under study were extracted and formatted in bulk using TBtools-II software.

The conversion of motif files from “.transfac” format to “.meme” format was performed using the transfac2meme tool within the locally installed MEME Suite software (Linux, version 5.5.1). This prepared the motifs for subsequent scanning of cis-acting elements. Scanning of cis-acting elements scanning was performed by using the FIMO command within this suite, setting the *p*-value threshold to less than 1.0 × 10^−4^, to identify significant matches.

### 4.13. Determination of Total Lignin Content and Phloroglucinol Staining in Mature Fibers

The total lignin content in mature fibers was determined using the acetyl bromide method [53], with further improvements made based on the method of Gao et al. [54]. Cotton fibers devoid of solid impurities were pre-cleaned according to the procedure outlined by Ferrarese et al. [55] to eliminate the interference of soluble impurities on the results. Given that this material was in the form of intact fibers rather than powder, the method of squeezing the fibers was employed as a substitute for centrifugation to facilitate the replacement of reagents. The cleaned fibers were then thoroughly dried at 60 °C until attaining a constant weight. An accurate mass of 0.1 g of mature fiber was added to 5 mL of a 25% acetyl bromide solution (diluted with glacial acetic acid), and the mixture was gently shaken at 70 °C for 60 min before being cooled on ice. Then, 4.5 mL of 2 M NaOH and 1.5 mL of 3 M hydroxylamine hydrochloride were added sequentially and mixed well. The volume of the solution was brought up to 25 mL with glacial acetic acid, and the mixture was allowed to stand at room temperature for 30 min. The absorbance of the solution at 280 nm was measured using a BioPhotometer (Eppendorf, Hamburg, Germany). At least three replicates were established for each sample to determine the total lignin content. A standard curve for lignin content was plotted (using the same steps) with alkaline lignin (L8500, Solarbio, Beijing, China). The standard curve equation is given as y = 0.0098x + 0.0124, with an R^2^ value of 0.9999. Here, “y” represents the absorbance value at 280 nm for the sample under test, and “x” denotes the lignin concentration in μg/mL.

Mature fibers were stained with 2% phloroglucinol [56]. The phloroglucinol staining solution was freshly prepared by dissolving phloroglucinol in anhydrous ethanol to a concentration of 4%, to which an equal volume of concentrated hydrochloric acid was added to achieve a final concentration of 2%. An amount of 0.1 g of mature fiber was stained with 3 mL of the staining solution for 45 min to observe the degree of color change.

## Figures and Tables

**Table 1:** Statistical analysis of yield traits in the T_2_ generation of *GhMYB52 Like* knockout transgenic cotton.

Line	Boll Weight (g)	Boll Seed Count	Single-Boll Fiber Weight (g)	Seed Index (g)	Lint Index (g)
HM-1	4.01 ± 0.08	23.9 ± 0.4	1.77 ± 0.06	9.50 ± 0.25	7.49 ± 0.10
377	3.75 ± 0.11 *	22.4 ± 1.2	1.61 ± 0.05 *	9.37 ± 0.26	7.06 ± 0.15 ***
397	3.56 ± 0.17 *	22.3 ± 0.5 *	1.56 ± 0.07 *	8.89 ± 0.20 ***	6.96 ± 0.12 ***

HM-1, wild type; 377 and 397, *GhMYB52 Like* functional knockout mutant lines. Data are presented as means ± standard deviation. All measurements, except for the Seed Index and lint index—which are supported by nine replicates each—were conducted with three replicates. Asterisks indicate levels of statistical significance, where * denotes *p* < 0.05, and *** denotes *p* < 0.001.

**Table 2:** Fiber quality analysis of T_2_ generation *GhMYB52 Like* knockout transgenic cotton.

Line	Upper-Half Mean Length/mm	Uniformity Index/%	Strength /cN·tex^−1^	Micronaire Value	Breaking Elongation Rate/%
HM-1	27.7 ± 0.4	84.7 ± 0.6	31.1 ± 0.6	5.1 ± 0.1	5.5 ± 0.1
377	27.4 ± 0.2	85.2 ± 0.3	30.7 ± 1.0	4.9 ± 0.1	5.8 ± 0.3
397	28.0 ± 0.5	85.2 ± 0.6	30.9 ± 1.6	4.9 ± 0.0	6.3 ± 0.4 *

HM-1, wild type; 377 and 397, *GhMYB52 Like* functional knockout mutant lines. Data are presented as means ± standard deviation. Each measurement was conducted with a minimum of three replicates. Asterisks indicate the level of statistical significance, with * denoting *p* < 0.05.

## Data Availability

The data presented in this study are available on request from the corresponding author due to privacy.

## Supplementary Materials

The following supporting information can be downloaded at https://www.mdpi.com/article/10.3390/ijms25094921/s1.

## References

[1] Kumar M., Campbell L., Turner S. (2016). Secondary cell walls: Biosynthesis and manipulation. J. Exp. Bot..

[2] Zhang B., Gao Y., Zhang L., Zhou Y. (2021). The plant cell wall: Biosynthesis, construction, and functions. J. Integr. Plant Biol..

[3] Pedersen G.B., Blaschek L., Frandsen K.E.H., Noack L.C., Persson S. (2023). Cellulose synthesis in land plants. Mol. Plant.

[4] Brandon A.G., Scheller H.V. (2020). Engineering of Bioenergy Crops: Dominant Genetic Approaches to Improve Polysaccharide Properties and Composition in Biomass. Front. Plant Sci..

[5] Carpita N.C., McCann M.C. (2020). Redesigning plant cell walls for the biomass-based bioeconomy. J. Biol. Chem..

[6] Dubos C., Stracke R., Grotewold E., Weisshaar B., Martin C., Lepiniec L. (2010). MYB transcription factors in *Arabidopsis*. Trends Plant Sci..

[7] Wu Y., Wen J., Xia Y., Zhang L., Du H. (2022). Evolution and functional diversification of R2R3-MYB transcription factors in plants. Hortic. Res..

[8] Jiang C.-K., Rao G.-Y. (2020). Insights into the Diversification and Evolution of R2R3-MYB Transcription Factors in Plants. Plant Physiol..

[9] Wei Z., Wei H. (2024). Deciphering the intricate hierarchical gene regulatory network: Unraveling multi-level regulation and modifications driving secondary cell wall formation. Hortic. Res..

[10] Kim H.J., Triplett B.A. (2001). Cotton Fiber Growth in Planta and in Vitro. Models for Plant Cell Elongation and Cell Wall Biogenesis. Plant Physiol..

[11] Haigler C.H., Betancur L., Stiff M.R., Tuttle J.R. (2012). Cotton fiber: A powerful single-cell model for cell wall and cellulose research. Front. Plant Sci..

[12] Basra A.S., Malik C.P. (1984). Development of the Cotton Fiber. International Review of Cytology.

[13] Khan M.A., Wahid A., Ahmad M., Tahir M.T., Ahmed M., Ahmad S., Hasanuzzaman M., Ahmad S., Hasanuzzaman M. (2020). World Cotton Production and Consumption: An Overview. Cotton Production and Uses: Agronomy, Crop Protection, and Postharvest Technologies.

[14] Fang S., Shang X., Yao Y., Li W., Guo W. (2020). NST-and SND-subgroup NAC proteins coordinately act to regulate secondary cell wall formation in cotton. Plant Sci..

[15] Ko J.H., Kim W.C., Han K.H. (2009). Ectopic expression of MYB46 identifies transcriptional regulatory genes involved in secondary wall biosynthesis in *Arabidopsis*. Plant J..

[16] McCarthy R.L., Zhong R., Ye Z.-H. (2009). MYB83 Is a Direct Target of SND1 and Acts Redundantly with MYB46 in the Regulation of Secondary Cell Wall Biosynthesis in *Arabidopsis*. Plant Cell Physiol..

[17] Xu X., Guerriero G., Berni R., Sergeant K., Guignard C., Lenouvel A., Hausman J.-F., Legay S. (2022). MdMYB52 regulates lignin biosynthesis upon the suberization process in apple. Front. Plant Sci..

[18] Cassan-Wang H., Goué N., Saidi M.N., Legay S., Sivadon P., Goffner D., Grima-Pettenati J. (2013). Identification of novel transcription factors regulating secondary cell wall formation in *Arabidopsis*. Front. Plant Sci..

[19] Chai G., Wang Z., Tang X., Yu L., Qi G., Wang D., Yan X., Kong Y., Zhou G. (2014). R2R3-MYB gene pairs in *Populus*: Evolution and contribution to secondary wall formation and flowering time. J. Exp. Bot..

[20] Fan L., Shi W.J., Hu W.R., Hao X.Y., Wang D.M., Yuan H., Yan H.Y. (2009). Molecular and Biochemical Evidence for Phenylpropanoid Synthesis and Presence of Wall-linked Phenolics in Cotton Fibers. J. Integr. Plant Biol..

[21] Wen X., Zhai Y., Zhang L., Chen Y., Zhu Z., Chen G., Wang K., Zhu Y. (2022). Molecular studies of cellulose synthase supercomplex from cotton fiber reveal its unique biochemical properties. Sci. China Life Sci..

[22] Kang L., Teng Y., Cen Q., Fang Y., Tian Q., Zhang X., Wang H., Zhang X., Xue D. (2022). Genome-Wide Identification of R2R3-MYB Transcription Factor and Expression Analysis under Abiotic Stress in Rice. Plants.

[23] Wilkins O., Nahal H., Foong J., Provart N.J., Campbell M.M. (2009). Expansion and Diversification of the Populus R2R3-MYB Family of Transcription Factors. Plant Physiol..

[24] Du H., Yang S.-S., Liang Z., Feng B.-R., Liu L., Huang Y.-B., Tang Y.-X. (2012). Genome-wide analysis of the MYB transcription factor superfamily in soybean. BMC Plant Biol..

[25] Zhong R., Ye Z.-H. (2014). Complexity of the transcriptional network controlling secondary wall biosynthesis. Plant Sci..

[26] Zhu Y., Li L. (2021). Multi-layered Regulation of Plant Cell Wall Thickening. Plant Cell Physiol..

[27] Ko J.-H., Jeon H.-W., Kim W.-C., Kim J.-Y., Han K.-H. (2014). The MYB46/MYB83-mediated transcriptional regulatory programme is a gatekeeper of secondary wall biosynthesis. Ann. Bot..

[28] Zhong R., Lee C., Zhou J., McCarthy R.L., Ye Z.-H. (2008). A Battery of Transcription Factors Involved in the Regulation of Secondary Cell Wall Biosynthesis in *Arabidopsis*. Plant Cell.

[29] Zhong R., Richardson E.A., Ye Z.-H. (2007). The MYB46 Transcription Factor Is a Direct Target of SND1 and Regulates Secondary Wall Biosynthesis in *Arabidopsis*. Plant Cell.

[30] Carroll A., Mansoori N., Li S., Lei L., Vernhettes S., Visser R.G., Somerville C., Gu Y., Trindade L.M. (2012). Complexes with Mixed Primary and Secondary Cellulose Synthases Are Functional in *Arabidopsis* Plants. Plant Physiol..

[31] Du J. (2019). Cloning and Function Analysis of the *MYB52* Transcription Factor Gene from *Gossypium hirsutum* L.. Master′s Thesis.

[32] Whetten R., Sederoff R. (1995). Lignin biosynthesis. Plant Cell.

[33] Xiao R., Zhang C., Guo X., Li H., Lu H. (2021). MYB Transcription Factors and Its Regulation in Secondary Cell Wall Formation and Lignin Biosynthesis during Xylem Development. Int. J. Mol. Sci..

[34] Zhou J., Lee C., Zhong R., Ye Z.-H. (2009). MYB58 and MYB63 Are Transcriptional Activators of the Lignin Biosynthetic Pathway during Secondary Cell Wall Formation in *Arabidopsis*. Plant Cell.

[35] Chen Z., Li Y., Teng Z., Zhang Y., Liu Y., Suo Q., Wang Y., Zeng J., Liang A., Yan Q. (2023). Cotton green fiber promotes suberin synthesis interfering cellulose deposition in the secondary cell wall. Ind. Crops Prod..

[36] Raes J., Rohde A., Christensen J.H., Van de Peer Y., Boerjan W. (2003). Genome-Wide Characterization of the Lignification Toolbox in *Arabidopsis*. Plant Physiol..

[37] Wang H.-Z., Dixon R.A. (2012). On–Off Switches for Secondary Cell Wall Biosynthesis. Mol. Plant.

[38] Zhong R., Ye Z.-H. (2012). MYB46 and MYB83 Bind to the SMRE Sites and Directly Activate a Suite of Transcription Factors and Secondary Wall Biosynthetic Genes. Plant Cell Physiol..

[39] Chen K., Song M., Guo Y., Liu L., Xue H., Dai H., Zhang Z. (2019). MdMYB46 could enhance salt and osmotic stress tolerance in apple by directly activating stress-responsive signals. Plant Biotechnol. J..

[40] Li Z., Su Q., Xu M., You J., Khan A.Q., Li J., Zhang X., Tu L., You C. (2020). Phenylpropanoid metabolism and pigmentation show divergent patterns between brown color and green color cottons as revealed by metabolic and gene expression analyses. J. Cotton Res..

[41] Luo M., Xiao Y., Li X., Lu X., Deng W., Li D., Hou L., Hu M., Li Y., Pei Y. (2007). GhDET2, a steroid 5α-reductase, plays an important role in cotton fiber cell initiation and elongation. Plant J..

[42] Wang P., Zhang J., Sun L., Ma Y., Xu J., Liang S., Deng J., Tan J., Zhang Q., Tu L. (2018). High efficient multisites genome editing in allotetraploid cotton (*Gossypium hirsutum*) using CRISPR/Cas9 system. Plant Biotechnol. J..

[43] Lei Y., Lu L., Liu H.-Y., Li S., Xing F., Chen L.-L. (2014). CRISPR-P: A Web Tool for Synthetic Single-Guide RNA Design of CRISPR-System in Plants. Mol. Plant.

[44] Xie K., Minkenberg B., Yang Y. (2015). Boosting CRISPR/Cas9 multiplex editing capability with the endogenous tRNA-processing system. Proc. Natl. Acad. Sci. USA.

[45] Zhang T., Hu Y., Jiang W., Fang L., Guan X., Chen J., Zhang J., Saski C.A., Scheffler B.E., Stelly D.M. (2015). Sequencing of allotetraploid cotton (*Gossypium hirsutum* L. acc. TM-1) provides a resource for fiber improvement. Nat. Biotechnol..

[46] Chen C., Wu Y., Li J., Wang X., Zeng Z., Xu J., Liu Y., Feng J., Chen H., He Y. (2023). TBtools-II: A “one for all, all for one” bioinformatics platform for biological big-data mining. Mol. Plant.

[47] Jumper J., Evans R., Pritzel A., Green T., Figurnov M., Ronneberger O., Tunyasuvunakool K., Bates R., Žídek A., Potapenko A. (2021). Highly accurate protein structure prediction with AlphaFold. Nature.

[48] Xie J., Chen Y., Cai G., Cai R., Hu Z., Wang H. (2023). Tree Visualization By One Table (tvBOT): A web application for visualizing, modifying and annotating phylogenetic trees. Nucleic Acids Res..

[49] Liu W., Xie X., Ma X., Li J., Chen J., Liu Y.-G. (2015). DSDecode: A Web-Based Tool for Decoding of Sequencing Chromatograms for Genotyping of Targeted Mutations. Mol. Plant.

[50] Sparkes I.A., Runions J., Kearns A., Hawes C. (2006). Rapid, transient expression of fluorescent fusion proteins in tobacco plants and generation of stably transformed plants. Nat. Protoc..

[51] (2006). Test Method of Properties of Cotton Fibers by High Volume Instruments.

[52] Hellens R.P., Allan A.C., Friel E.N., Bolitho K., Grafton K., Templeton M.D., Karunairetnam S., Gleave A.P., Laing W.A. (2005). Transient expression vectors for functional genomics, quantification of promoter activity and RNA silencing in plants. Plant Methods.

[53] Moreira-Vilar F.C., Siqueira-Soares R.d.C., Finger-Teixeira A., Oliveira D.M.d., Ferro A.P., da Rocha G.J., Ferrarese M.d.L.L., dos Santos W.D., Ferrarese-Filho O. (2014). The Acetyl Bromide Method Is Faster, Simpler and Presents Best Recovery of Lignin in Different Herbaceous Tissues than Klason and Thioglycolic Acid Methods. PLoS ONE.

[54] Gao Z., Sun W., Wang J., Zhao C., Zuo K. (2019). GhbHLH18 negatively regulates fiber strength and length by enhancing lignin biosynthesis in cotton fibers. Plant Sci..

[55] Ferrarese M.L., Zottis A., Ferrarese-Filho O. (2002). Protein-free lignin quantification in soybean (*Glycine max*) roots. Sect. Bot..

[56] Han L.-B., Li Y.-B., Wang H.-Y., Wu X.-M., Li C.-L., Luo M., Wu S.-J., Kong Z.-S., Pei Y., Jiao G.-L. (2013). The Dual Functions of WLIM1a in Cell Elongation and Secondary Wall Formation in Developing Cotton Fibers. Plant Cell.

