# Supplementary material for: GhMYB52 Like: A Key Factor That Enhances Lint Yield by Negatively Regulating the Lignin Biosynthesis Pathway in Fibers of Upland Cotton (Gossypium hirsutum L.)
Source: Int J Mol Sci. 2024 Apr 30;25(9):4921. doi: 10.3390/ijms25094921 (PMC11084151; doi:10.3390/ijms25094921)
Supplement: Supplementary file 1 [file ijms-25-04921-s001.zip › ijms-2944568-supplementary.pdf]

Supplementary materials: Figures

GhMYB52 Like: A Key Factor that Enhances Lint Yield by Negatively Regulating the Lignin Biosynthesis Pathway in Fibers of Upland Cotton (*Gossypium hirsutum* L.)

Yang Yang [1,2], Xue Zhou [3], Xi Zhu [1,2], Bo Ding [1,2], Linzhu Jiang [1,2], Huiming Zhang [1,2], Silu Li [1,2], Shuyan Cao [1,2], Mi Zhang [1,2], Yan Pei [1,2] and Lei Hou [1,2,*]

1 College of Agronomy and Biotechnology, Southwest University, Chongqing 400715, China; neo5334@163.com (Y.Y.); zhuxi0603@email.swu.edu.cn (X.Z.); 15086655435@163.com (B.D.); linzhuhh@163.com (L.J.); zhang15828612304@163.com (H.Z.); lisilu20010817@outlook.com (S.L.); shuyan961029@163.com (S.C.); selenazm@swu.edu.cn (M.Z.); peiyan3@swu.edu.cn (Y.P.)

2 Chongqing Key Laboratory of Application and Safety Control of Genetically Modified Crops, Southwest University, Chongqing 400715, China

3 Laboratory Animal Center, Southwest University, Chongqing 400715, China; yokie01@swu.edu.cn

* Correspondence: houlei@swu.edu.cn; Tel./Fax: +86-023-68251264

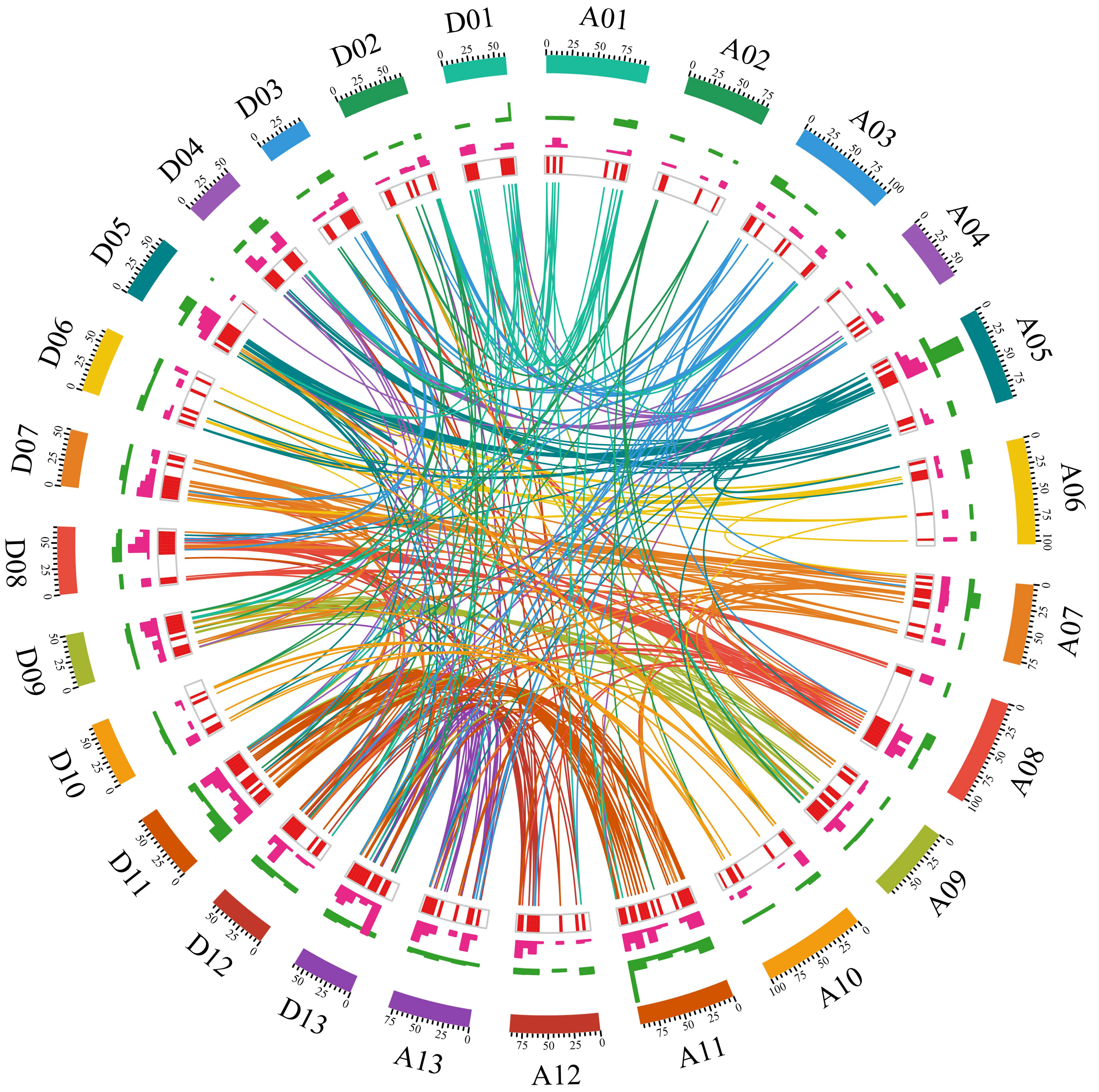

Figure S1: Collinearity Analysis of R2R3 MYB Family in *Gossypium hirsutum.* From the outside in: Chromosomes are arranged in the outermost layer as numbered circular rectangles, with scale markings indicating chromosome length (unit: Mb). A green bar graph layer displays the FPKM values of R2R3 MYBs in fiber cells at 20DPA. A rose-red bar graph layer shows the amino acid length data of R2R3 MYBs proteins. Rectangles with red lines indicate the density and location of R2R3 MYBs genes on each chromosome. The innermost layer of colored lines connects homologous genes within the R2R3 MYB family.

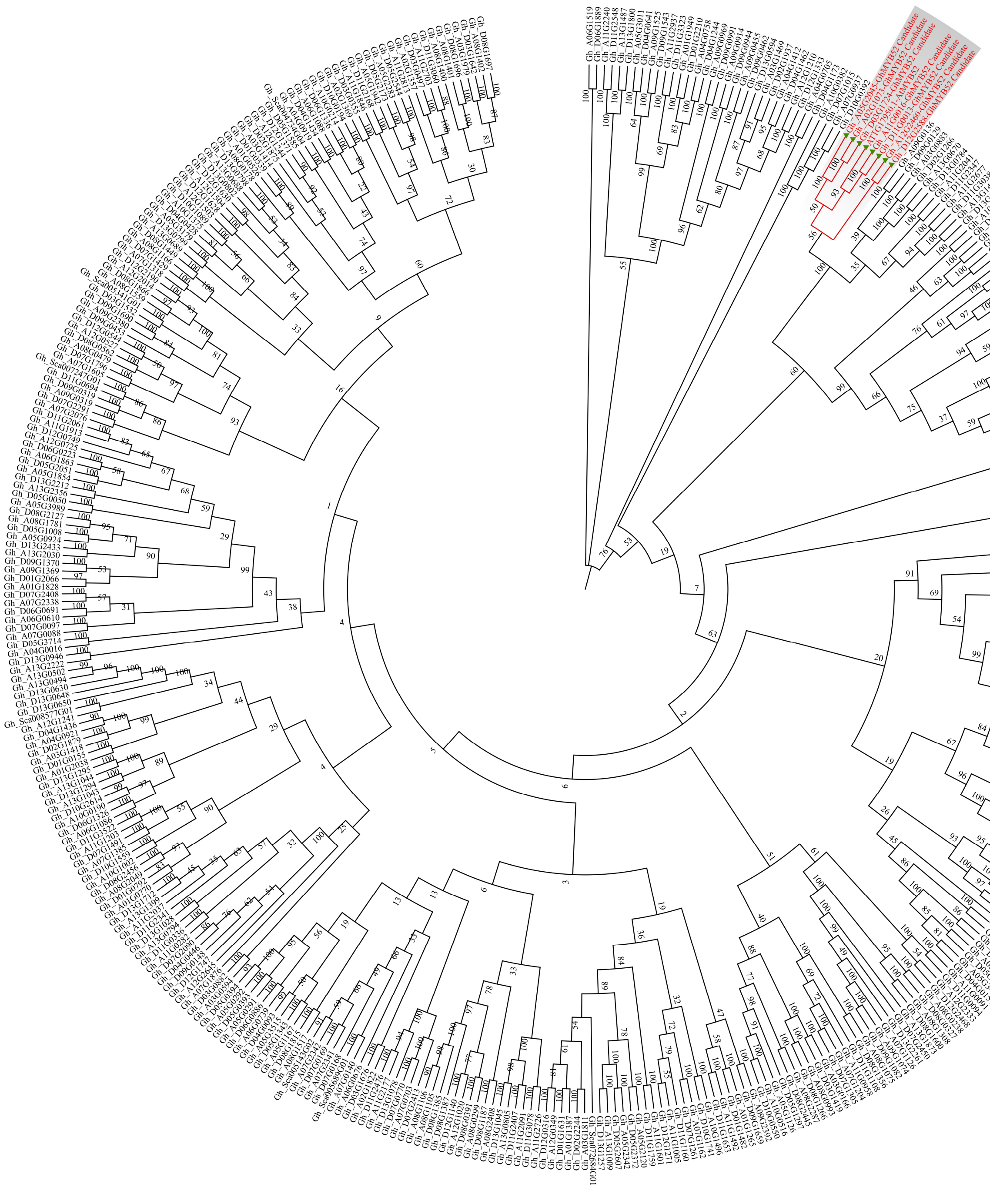

Figure S2: Evolution and Diversity of R2R3 MYB Proteins in *Gossypium hirsutum* Compared to *Arabidopsis* AtMYB52. The phylogenetic tree depicts the evolutionary interrelations among 414 R2R3 MYB proteins identified in *Gossypium hirsutum*, utilizing the AtMYB52 protein from *Arabidopsis thaliana* as an external reference point. The red font highlights seven R2R3 MYB proteins highly homologous to AtMYB52 within upland cotton. The amino acid sequences of the two species were analyzed using the MEGA software (version 11.0.13) employing the Neighbor-Joining method, with 1000 bootstrap replicates for tree construction. Bootstrap support values are presented as percentages.

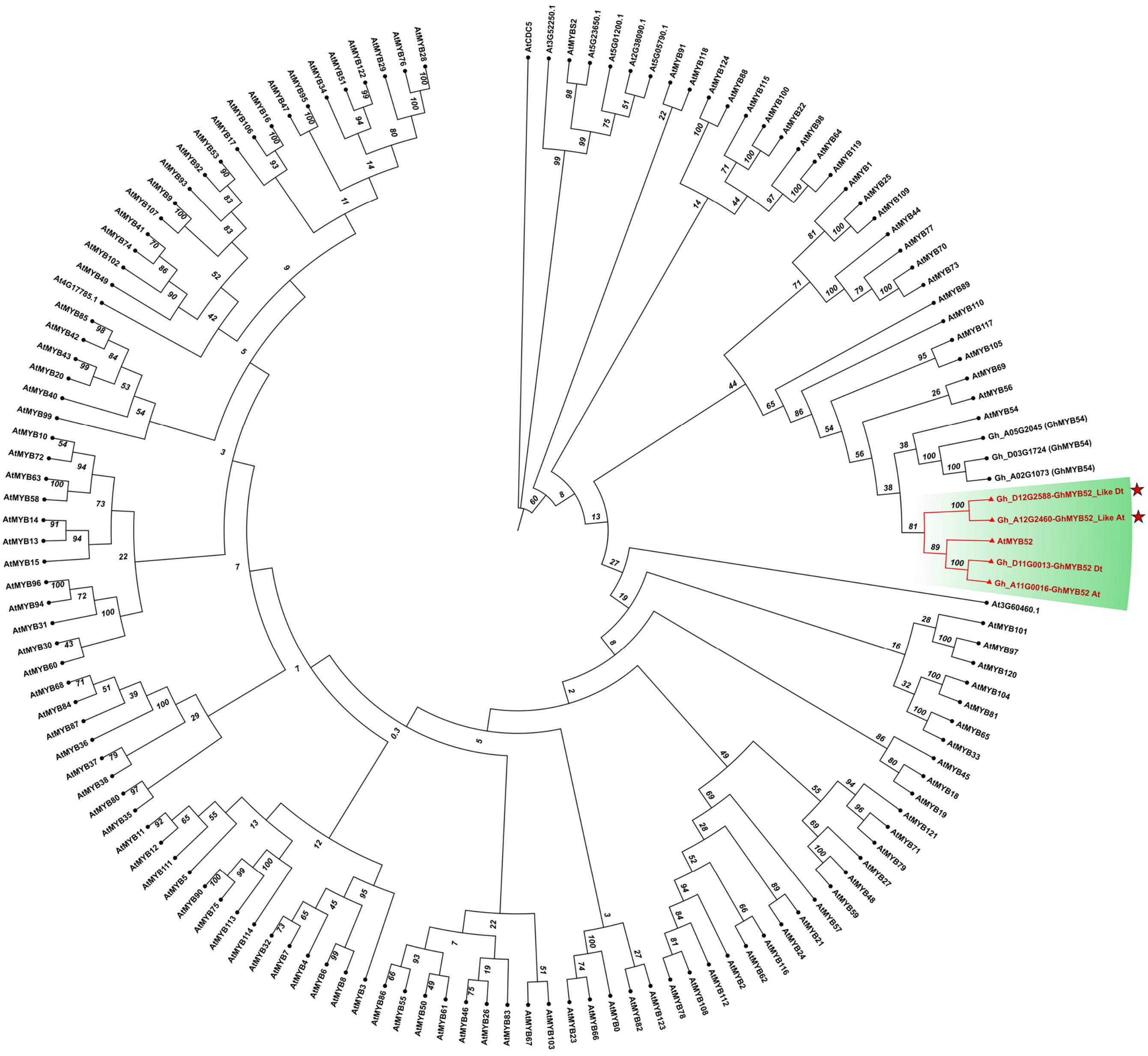

Figure S3: Phylogenetic Analysis of *Arabidopsis thaliana* R2R3 MYB Proteins with *Gossypium hirsutum* GhMYB52 Candidates. This phylogenetic tree encompasses all 133 R2R3 MYB family proteins from *Arabidopsis thaliana* alongside 7 GhMYB52 candidate proteins from *Gossypium hirsutum*, specifically aiming to elucidate the evolutionary relationships between GhMYB52 and GhMYB52 Like proteins. At and Dt refer to the A and D sub-genomes of upland cotton, respectively. The amino acid sequences of the two species were analyzed using the MEGA software (version 11.0.13) employing the Neighbor-Joining method, with 1000 bootstrap replicates for tree construction. Bootstrap support values are presented as percentages.

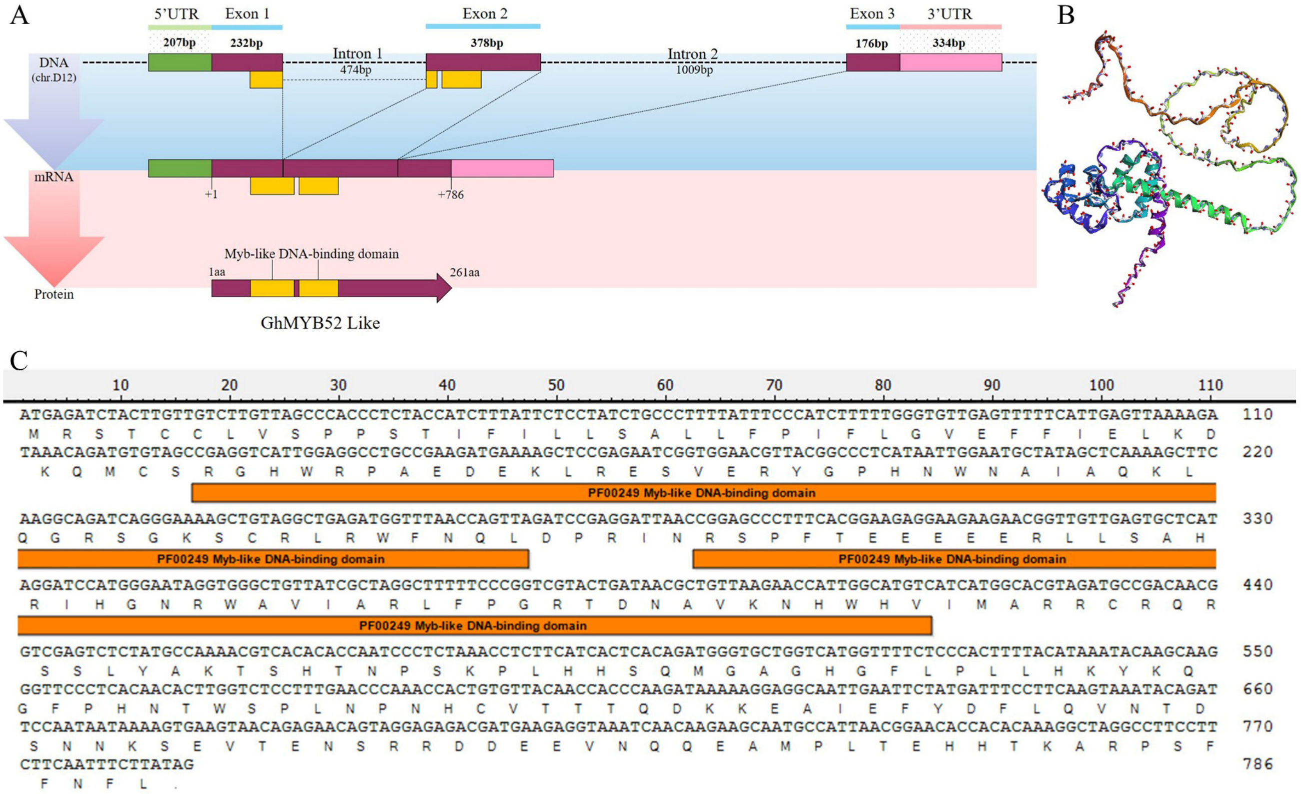

Figure S4: Structural Insights into *GhMYB52 Like* Gene Expression and Protein Architecture. A) Schematic of *GhMYB52 Like* Transcription and Translation Processes. This panel highlights the transcription of *Gh_D12G2588* on chromosome D12 into mRNA, detailing the location, length, and conserved domains of the mRNA region. B) Tertiary Structure Prediction of GhMYB52 Like. The three-dimensional structure of GhMYB52 Like, predicted using the deep convolutional neural network model AlphaFold2, exhibits the characteristic Helix-Turn-Helix (HTH) structural motif typical of R2R3 MYBs. C) Nucleotide and Amino Acid Sequences of the *Gh_D12G2588* Coding Region. The coding sequence (CDS) spans 786 base pairs, encoding 261 amino acids.

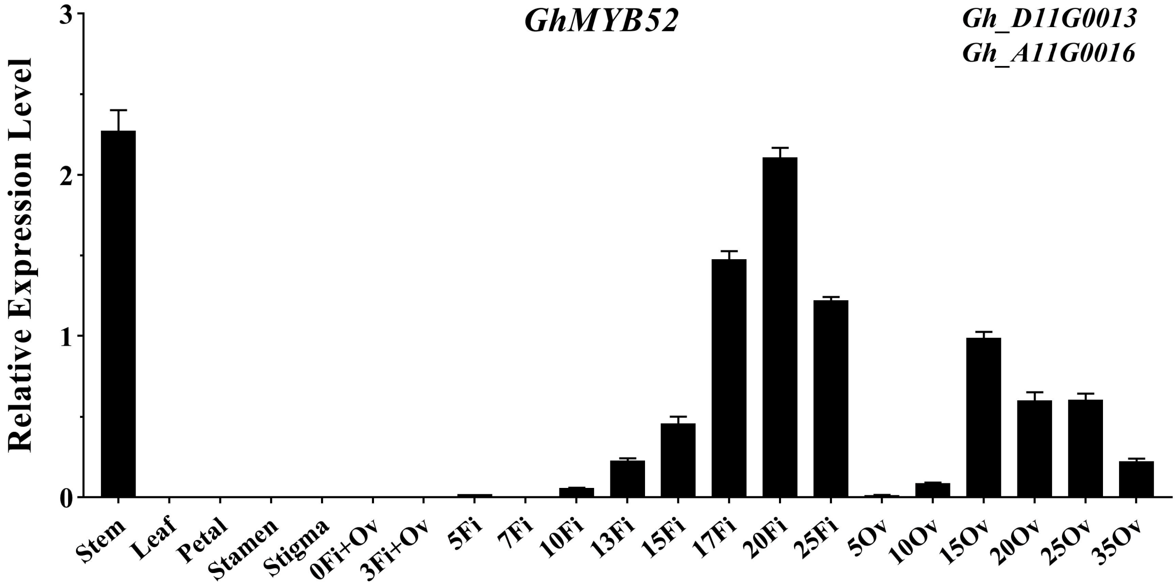

Figure S5: Expression Analysis of *GhMYB52* in Multiple Tissues of Wild-Type *Gossypium hirsutum* HM-1 Cultivar. This figure presents the expression patterns of *GhMYB52* across 21 different tissue types in the HM-1 cultivar of upland cotton. Numerical values next to tissue types represent days post-anthesis. 'Fi' represents fibers, 'Ov' signifies ovules, and 'Fi+Ov' denotes ovules with fibers not separated. Specific primers were employed for the simultaneous amplification of transcripts from both *Gh_A11G0016* and *Gh_D11G0013*. *GhHistone3* was utilized as an internal reference gene, with each experiment conducted in triplicate. The data underscore that *GhMYB52* does not exhibit fiber-specific expression.

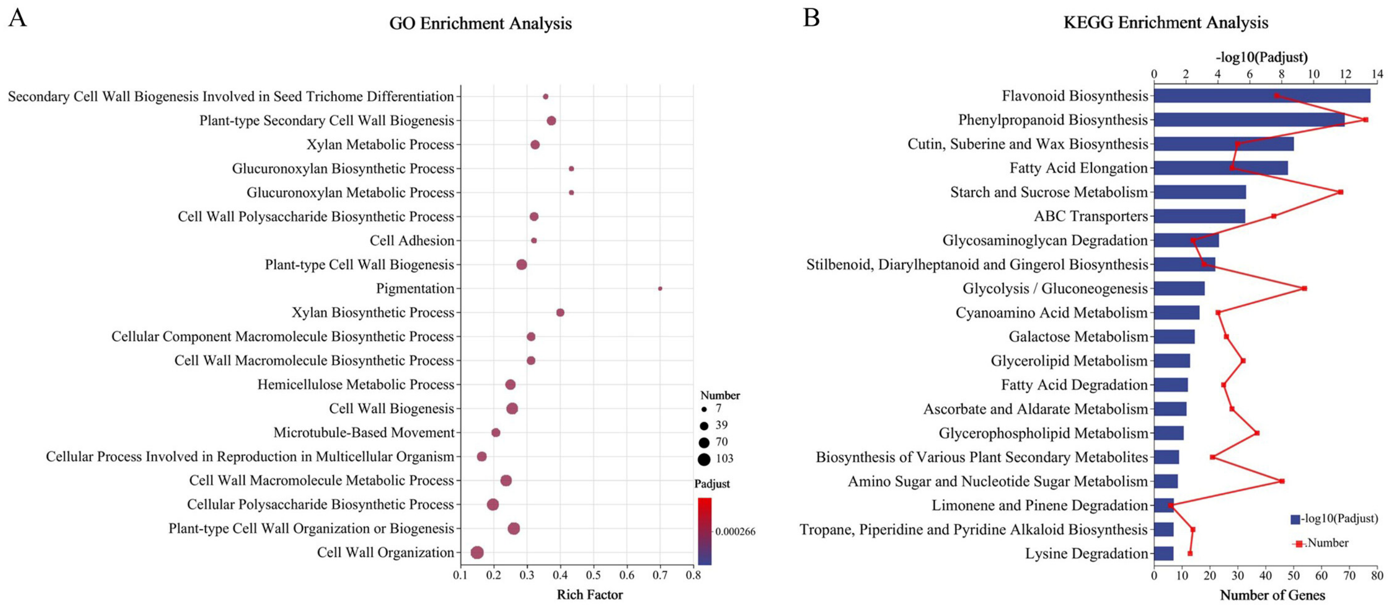

Figure S6: Transcriptome-Wide Enrichment Analysis of Differentially Expressed Genes in 16DPA Cotton Fibers (HM-1 versus 377). Differential gene expression was analyzed using DESeq2, applying a significance threshold of adjusted p < 0.05 and a minimum fold change of 10. A) Gene Ontology (GO) enrichment analysis of differentially expressed genes in 16DPA cotton fiber samples. The size of the bubbles represents the number of genes enriched within each GO term. B) Kyoto Encyclopedia of Genes and Genomes (KEGG) pathway enrichment analysis of differentially expressed genes in 16DPA cotton fiber samples. The red line indicates the number of genes involved in each pathway, while the blue bars represent the -log10(Padjust) values, signifying the statistical significance of the enrichment. Both subfigures showcase the top 20 results from the enrichment analysis. Together, these analyses afford an integrated overview of the transcriptional changes in fibers from the *GhMYB52 Like* mutant, highlighting the pathways that may be modulated by the *GhMYB52 Lik*e gene in cotton fiber development.

Gene	Fiber 05D	Fiber 10D	Fiber 20D	Fiber 25D	Ovule -3D	Ovule -1D	Ovule 00D	Ovule 01D	Ovule 03D	Ovule 05D	Ovule 10D	Ovule 20D	Ovule 25D	Ovule 35D
Gh_A05G3959 - GhCesA1A_A	18.19	19.13	13.03	13.01	8.07	4.90	6.11	5.30	9.00	3.77	12.70	16.23	9.05	3.97
Gh_D05G0084 - GhCesA1A_D	17.35	23.03	23.12	18.23	11.74	8.74	9.07	6.24	11.53	8.72	31.63	29.46	11.04	2.99
Gh_A05G3967 - GhCesA1B_A	37.43	52.91	41.05	31.95	16.94	15.54	18.32	16.91	19.77	8.56	35.33	31.25	20.73	12.27
Gh_D05G0077 - GhCesA1B_D	40.26	51.22	26.47	25.63	11.10	7.37	9.95	9.22	16.16	6.35	31.59	23.12	15.88	6.11
Gh_A08G0498 - GhCesA3A_A	3.16	3.43	11.62	7.80	6.08	10.03	11.22	6.28	4.81	4.75	8.08	10.03	9.13	4.12
Gh_D08G0584 - GhCesA3A_D	26.99	27.69	44.86	34.96	21.67	20.75	17.67	27.71	24.60	23.60	45.74	31.35	21.04	8.95
Gh_A08G1305 - GhCesA3B_A	52.36	50.95	29.37	27.10	25.62	23.30	22.66	25.15	31.25	31.70	44.17	30.42	17.62	7.33
Gh_D08G1597 - GhCesA3B_D	42.79	42.12	29.59	19.87	23.43	25.21	23.39	22.42	25.84	28.19	36.44	21.79	13.06	6.33
Gh_A02G1066 - GhCesA3C_A	1.27	1.42	2.38	3.34	3.11	1.01	2.88	1.38	2.16	0.65	3.80	6.40	4.12	2.55
Gh_D03G0611 - GhCesA3C_D	1.73	1.33	2.58	4.60	2.90	1.63	2.01	1.73	2.55	1.10	4.09	4.49	3.41	2.23
Gh_A02G1317 - GhCesA6A_A	9.29	4.59	8.78	20.87	25.31	12.76	18.15	12.06	14.71	5.80	25.01	9.87	20.48	5.67
Gh_D03G0455 - GhCesA6A_D	7.17	5.71	11.65	18.38	20.80	8.70	22.45	7.97	12.45	4.84	29.72	6.24	17.95	2.95
Gh_A05G3694 - GhCesA6B_A	24.41	16.23	38.11	42.99	9.60	23.50	16.40	15.54	18.16	20.10	28.48	50.01	28.24	8.44
Gh_D05G2313 - GhCesA6B_D	9.41	3.22	20.80	30.39	12.77	19.43	13.85	13.60	12.84	17.20	25.98	41.16	23.34	7.69
Gh_A06G1017 - GhCesA6C_A	17.59	11.91	7.94	7.63	6.74	5.20	5.93	3.85	8.99	4.76	11.46	8.47	5.73	2.78
Gh_D06G1219 - GhCesA6C_D	8.57	3.74	8.09	6.14	4.97	3.84	4.11	2.26	5.13	2.99	8.90	5.46	4.25	2.27
Gh_A11G3209 - GhCesA6D_A	7.66	9.51	9.24	11.12	7.94	5.48	7.52	3.97	6.70	3.94	20.99	7.73	7.33	4.01
Gh_D11G2235 - GhCesA6D_D	4.19	6.69	3.66	7.63	7.36	7.36	5.88	6.31	5.77	3.30	12.24	5.58	7.54	6.42
Gh_D12G0885 - GhCesA6_D12	3.96	2.44	1.48	1.98	4.61	1.41	3.49	1.34	2.56	0.91	4.17	1.55	1.28	0.89
Gh_A13G1378 - GhCesA6_A13	0.00	0.00	0.00	0.11	0.00	0.00	0.00	0.00	0.00	0.00	0.00	0.00	0.00	0.00
Gh_D13G1693 - GhCesA6_D13	0.00	0.00	0.00	0.02	0.00	0.00	0.00	0.00	0.00	0.00	0.00	0.00	0.00	0.00
Gh_A07G1871 - GhCesA4A_A	0.15	0.10	109.62	36.60	0.00	0.03	0.21	0.02	0.07	0.06	0.61	8.86	3.14	0.29
Gh_D07G2083 - GhCesA4A_D	0.12	0.07	103.66	40.84	0.00	0.03	0.05	0.09	0.01	0.23	0.66	18.74	4.85	0.29
Gh_A08G0421 - GhCesA4B_A	1.69	1.43	347.57	171.98	1.66	1.89	1.59	1.45	1.18	2.73	2.94	46.46	8.87	0.80
Gh_D08G0509 - GhCesA4B_D	3.12	3.31	341.37	161.51	3.35	2.40	2.09	2.19	2.26	2.19	4.00	47.88	9.42	1.12
Gh_A07G0322 - GhCesA7A_A	0.03	0.00	1.22	0.79	0.00	0.04	0.00	0.00	0.02	0.06	0.00	0.51	0.39	0.00
Gh_D07G0380 - GhCesA7A_D	0.41	0.14	224.88	127.45	0.05	0.14	0.10	0.07	0.10	0.11	1.16	36.93	9.18	0.34
Gh_A05G3965 - GhCesA7B_A	0.41	0.10	394.32	163.47	0.05	0.17	0.36	0.11	0.03	0.39	0.71	41.53	8.39	0.05
Gh_D05G0079 - GhCesA7B_D	0.44	0.07	416.65	182.43	0.10	0.24	0.33	0.14	0.10	0.84	1.47	54.51	11.95	0.39
Gh_D05G1460 - GhCesA8A_D	1.44	0.94	2.06	1.97	4.62	1.74	3.20	3.66	2.40	2.67	3.12	8.34	1.47	0.57
Gh_A10G0327 - GhCesA8B_A	0.70	0.25	378.86	180.99	0.17	0.19	0.38	0.32	0.27	0.96	2.00	53.82	14.57	0.41
Gh_D10G0333 - GhCesA8B_D	0.59	0.69	381.80	184.35	0.94	0.37	0.52	0.37	0.47	0.78	4.38	68.10	13.94	0.52

Color scale: 0.0 – 0.2 – 0.4 – 0.6 – 0.8 – 1.0

Figure S7: Expression Heatmap of the Cellulose Synthase (*GhCesA*) Gene Family During Fiber and Ovule Development in the *Gossypium hirsutum* TM-1 Cultivar. Each row represents an individual *GhCesA* gene, while each column corresponds to different tissue types. On the x-axis, positive integers denote days post-anthesis, while negative numbers indicate days before anthesis. The FPKM values of the *GhCesA* gene family are displayed in the grid for four time points in fiber tissues and ten time points in ovules. The intensity of the color represents the relative expression level, with red indicating higher expression and white indicating lower expression. The most intense red in each row highlights the tissue type where the corresponding gene is most highly expressed, but this does not necessarily reflect the highest absolute expression values when comparing across different genes (for detailed FPKM values, refer to the data).

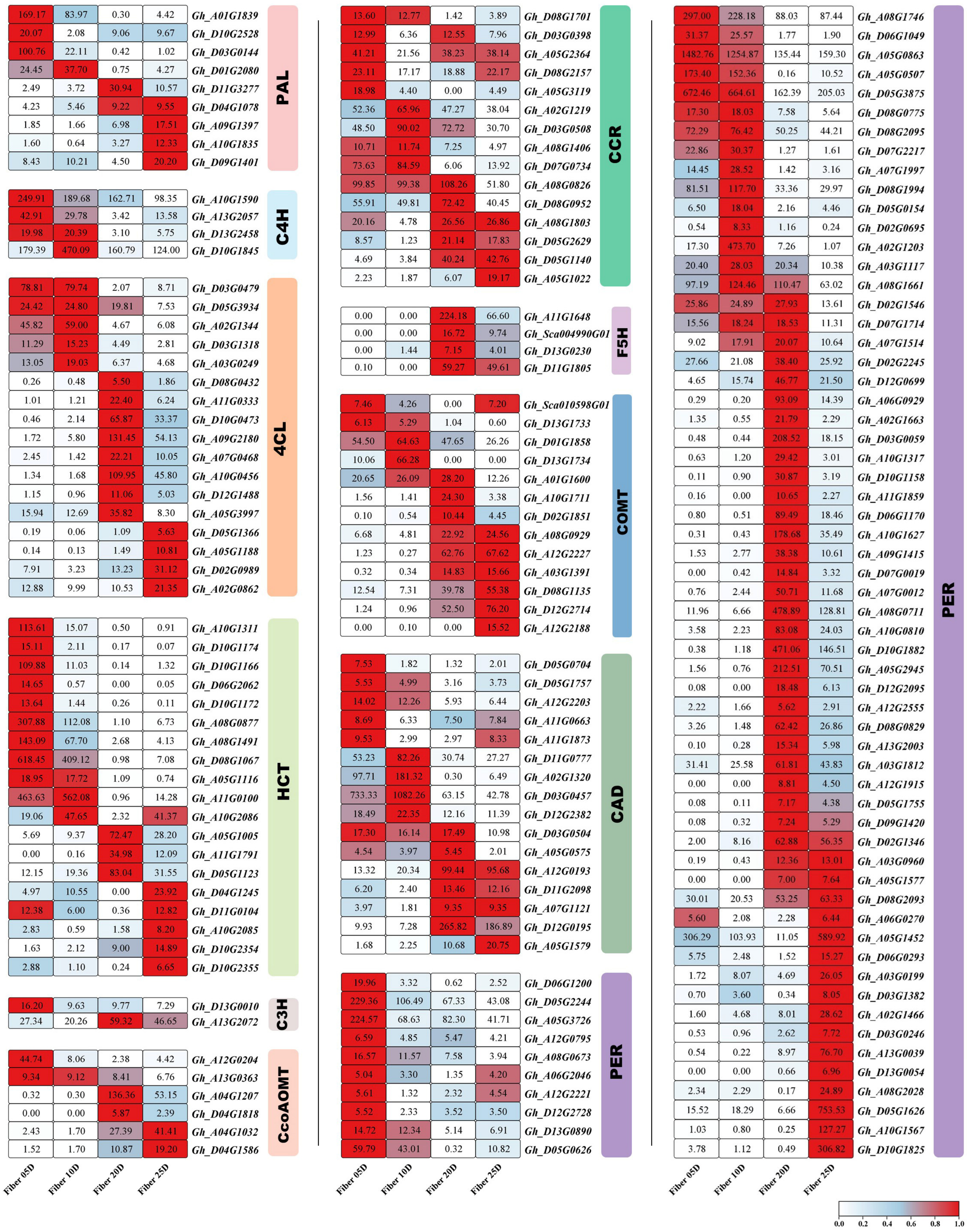

Figure S8: Expression Heatmap of Genes Related to Lignin Biosynthesis During Fiber Development in the *Gossypium hirsutum* TM-1 Cultivar. The heatmap displays only those genes within each category where at least one tissue type has an FPKM value greater than 5. Each row represents an individual gene, and each column represents different tissue types. On the x-axis, positive integers denote days post-anthesis. The FPKM values for lignin biosynthesis-related genes at four time points in fiber tissues are shown in

the grid. The intensity of the color represents the relative expression level, with red indicating higher expression and white indicating lower expression. The most intense red in each row highlights the tissue type where the corresponding gene is most highly expressed, but this does not necessarily reflect the highest absolute expression values when comparing across different genes (for detailed FPKM values, refer to the data). PAL: Phenylalanine Ammonia-Lyase; C4H: Cinnamic Acid 4-Hydroxylase; 4CL: 4-Coumarate-Coenzyme A Ligase; HCT: Hydroxycinnamoyl Transferase; C3H: p-Coumarate 3-Hydroxylase; CcoAOMT: Caffeoyl Coenzyme A 3-O-Methyltransferase; CCR: Cinnamoyl-CoA Reductase; F5H: Ferulate 5-Hydroxylase; COMT: Caffeic Acid O-Methyltransferase; CAD: Cinnamyl Alcohol Dehydrogenase; PER: Peroxidase.
